# Metabolic relationships between marine red algae and algae-associated bacteria

**DOI:** 10.1007/s42995-024-00227-z

**Published:** 2024-05-08

**Authors:** Kyung Hyun Kim, Jeong Min Kim, Ju Hye Baek, Sang Eun Jeong, Hocheol Kim, Hwan Su Yoon, Che Ok Jeon

**Affiliations:** 1 Department of Biological Sciences and Biotechnology, Hannam University, Daejon, 34054 Republic of Korea; 2 Department of Life Science, Chung-Ang University, Seoul, 06974 Republic of Korea; 3 Department of Biological Sciences, Sungkyunkwan University, Suwon, 16419 Republic of Korea

**Keywords:** Metabolic relationship, Marine red algae, Symbiotic bacteria, *Roseibium*, *Phycisphaera*, *Porphyridium*

## Abstract

Mutualistic interactions between marine phototrophs and associated bacteria are an important strategy for their successful survival in the ocean, but little is known about their metabolic relationships. Here, bacterial communities in the algal sphere (AS) and bulk solution (BS) of nine marine red algal cultures were analyzed, and *Roseibium* and *Phycisphaera* were identified significantly more abundantly in AS than in BS. The metabolic features of *Roseibium* RMAR6-6 (isolated and genome-sequenced), *Phycisphaera* MAG 12 (obtained by metagenomic sequencing), and a marine red alga, *Porphyridium purpureum* CCMP1328 (from GenBank), were analyzed bioinformatically. RMAR6-6 has the genetic capability to fix nitrogen and produce B vitamins (B1, B2, B5, B6, B9, and B12), bacterioferritin, dimethylsulfoniopropionate (DMSP), and phenylacetate that may enhance algal growth, whereas MAG 12 may have a limited metabolic capability, not producing vitamins B9 and B12, DMSP, phenylacetate, and siderophores, but with the ability to produce bacitracin, possibly modulating algal microbiome. *P*. *purpureum* CCMP1328 lacks the genetic capability to fix nitrogen and produce vitamin B12, DMSP, phenylacetate, and siderophore. It was shown that the nitrogen-fixing ability of RMAR6-6 promoted the growth of *P*. *purpureum*, and DMSP reduced the oxidative stress of *P*. *purpureum*. The metabolic interactions between strain RMAR6-6 and *P*. *purpureum* CCMP1328 were also investigated by the transcriptomic analyses of their monoculture and co-culture. Taken together, potential metabolic relationships between *Roseibium* and *P*. *purpureum* were proposed. This study provides a better understanding of the metabolic relationships between marine algae and algae-associated bacteria for successful growth.

**Supplementary Information:**

The online version contains supplementary material available at 10.1007/s42995-024-00227-z.

## Introduction

Marine algae, photosynthetic eukaryotic organisms, are one of the major primary producers widely distributed in the ocean, and make significant contributions to the global cycle of nutrients, such as carbon, sulfur, nitrogen, and phosphorus (Buchan et al. 2014). Marine algae occur in close association with various heterotrophic bacteria that play critical roles in their growth and survival (Cirri and Pohnert 2019). Co-evolved symbiotic relationships between marine algae and algae-associated bacteria generally benefit marine algal growth and development, but they may also be neutral or harmful to the algal hosts depending on the bacteria (Amin et al. 2015; van Tol et al. 2017). For example, symbiotic bacteria can promote the growth of marine algae through the production of phytohormones, B vitamins, siderophore, or micro-nutrients (Amin et al. 2015; Croft et al. 2005; Wienhausen et al. 2017), whereas some bacteria may compete with marine algae for resources, cause diseases in algae, or produce algicidal compounds (Li et al. 2022; Seymour et al. 2017; van Tol et al. 2017). The proliferation or death of marine algae may even be caused by the same bacteria depending on the conditions (Segev et al. 2016; Seyedsayamdost et al. 2011). Global climate change has instigated many studies on microbe-algae interactions in marine algae, including algae in corals (because they are sensitive to temperature rise), calcareous phytoplankton, such as diatoms (for CO_2_ sinking), and dinoflagellates (Amin et al. 2015; Maire et al. 2021; Shibl et al. 2020; van Tol et al. 2017). Much of this research has focused mainly on identifying 'key microbes' associated with corals, diatoms, and dinoflagellates, and characterizing their symbiotic interactions that affect growth or survival (Ainsworth et al. 2015; Astudillo-García et al. 2017; Lawson et al. 2018). However, studies on the microbe-algae interactions in seaweeds, including red-, brown- and green algae, have received less attention despite the attractive value as food, food additives, and industrial resources, as well as their ecological importance in food webs and the global nutrient cycle (Barros et al. 2022; Dawczynski et al. 2007; Pacheco et al. 2020). Like other marine algae, seaweed growth or survival may also be closely related to interactions with their surrounding microbes (Egan et al. 2013; Ghaderiardakani et al. 2020; Singh and Reddy 2014), yet the association of heterotrophic bacteria with seaweeds has been rarely explored, and little is known about their metabolic interactions with seaweeds.

The phylum *Rhodophyta*, commonly known as red algae, encompasses over 6000 genetically diverse eukaryotic species that are widely distributed in the ocean (Yang et al. 2016). We collected nine cultures of marine microalgae and hypothesized the existence of common microbiota—a shared assemblage of microbes— even in marine red microalgae, influencing their survival and proliferation in oceanic environments. Our postulation posits that microbiota more tightly attached to marine algae, referred to as the microbiome within the algal sphere (AS; attached to algae) in this study, would exert a more direct impact on the growth of marine algae than microbiota in the bulk solution (BS; not tightly attached to marine algae) of algal cultures because the closer the distance to each other, the more active the exchange of metabolites. Accordingly in this study, we analyzed bacterial communities in both AS and BS of marine red algal cultures and compared their bacterial compositions statistically to identify microbiota associated with red algae. Furthermore, we explored the metabolic relationships of potentially symbiotic microbiota with *Porphyridium* (*P*.) *purpureum* CCMP1328, a marine red alga available as an axenic culture, through comprehensive genomic analysis, co-culture experiments, and transcriptomic studies.

## Materials and methods

### Collection of marine red algal cultures and culture conditions

Nine xenic marine red algal cultures were obtained from Prof. Hwan Su Yoon (Sungkyunkwan University, South Korea). Axenic bulk cultures of *P*. *purpureum* CCMP1328 were purchased from the National Center for Marine Algae and Microbiota at Bigelow Laboratory. Marine red algae were confirmed taxonomically by sequencing the *rbcL*, as described previously (Yang et al. 2016). The detailed information on algal cultures used in this study is described in Supplementary Table S1. Algal cultures were maintained under 80 ± 5 μmol photons m^−2^ s^−1^ (cool-white fluorescent lamps) with a 16/8 h light/dark cycle in 175 cm^2^ cell culture flasks (SPL Life Sciences, Korea) containing 100 mL of L1 medium (Guillard and Hargraves 1993) at 14 ℃ and subcultured by transferring algal cultures into fresh L1 medium (1:5) every month.

### Bacterial community analysis

Bacterial communities in the AS and BS of marine red algal cultures were analyzed and compared. To obtain AS and BS samples, approximately 50 mL of algal cultures were filtered using a 5 μm membrane filter (MilliporeSigma, USA) and washed twice with sterilized L1 medium. Bacterial cells in the filtrate were harvested by centrifugation (16,100×*g*, 10 min). Filtered cells on the membrane filters, and bacterial cells harvested from the filtrate, were used for the bacterial community analysis in AS and BS of marine red algae, respectively, and their genomic DNA was extracted using the FastDNA SPIN Kit (MP Biomedicals, USA), according to the manufacturer’s instructions. Hypervariable regions (V1–V3) of bacterial 16S rRNA genes were PCR-amplified and sequenced using a 454 GS-FLX Titanium system (Roche, Germany) at Macrogen (Korea), and bacterial communities were analyzed as described previously (Lee et al. 2018). To compare the bacterial community of seawater with a control, approximately 2 L of seawater sampled from the East Sea of South Korea was filtered through a 0.2 μm membrane filter (Merck Millipore, USA), and bacterial cell communities on the membrane filter were analyzed using an Illumina MiSeq platform (Roche, Germany), as described previously (Miran et al. 2017). Principal component analysis using the prcomp function in R software was performed to compare bacterial communities in seawater, BS, and SA at the genus level. Hierarchical clustering of bacterial communities in BS and AS was performed based on the weighted Euclidean distance and visualized as dendrograms using GENE-E software (http://www.broadinstitute.org/cancer/software/GENE-E/). Bacterial genera significantly differentiated in BS and AS were analyzed using the linear discriminant analysis effective size (LEfSe) algorithm with default parameters (Segata et al. 2011).

### Bacterial isolation and genome sequencing

To isolate strains belonging to bacterial genera with significantly higher abundances in AS than in BS, the xenic cultures of *As*. *taxiformis* and *Rhodo*. *marinus* were filtered through a 5 μm membrane filter. Filtered algal cells were homogenized in L1 medium using a homogenizer, spread on marine 2216E agar (MA; BD, USA), 1/5 × MA supplemented with 2% (w/v) NaCl, and L1 medium agar containing 0.3% pyruvate, and aerobically incubated at 20 °C. Colonies grown on agar media were classified phylogenetically, as described previously (Jeong et al. 2017). The genome of RMAR6-6 isolated from the *Rhodo*. *marinus* culture was completely sequenced using a combination of PacBio RS II SMRT (10 kb library) and Illumina HiSeq X (151 bp paired-end) sequencing, as described previously (Baek et al. 2020). Circular maps of the chromosome and plasmids of RMAR6-6 were generated using the integrated CGView server (http://cgview.ca/) (Stothard and Wishart 2005).

### Shotgun metagenome sequencing and assembly

The genomic information on uncultured bacteria with significantly higher abundances in AS than in BS was obtained through shotgun metagenome sequencing. Total genomic DNA was extracted from the filtered cells of the *Rhodo*. *marinus* culture and sequenced using an Illumina-MiSeq paired-end platform (× 300 bp) at Macrogen. Sequencing reads were trimmed (Q ≥ 30, length ≥ 100 nucleotides) using Sickle and de novo-assembled using SPAdes (Nurk et al. 2017) for k-mers of 21, 33, 55, 77, 99, and 127 nucleotides with the "–meta" option. The metagenome-assembled contigs were binned using DAS Tool (Sieber et al. 2018) with default parameters. The quality of MAGs obtained from the metagenomic binning was assessed using the CheckM program (Parks et al. 2015). Taxonomic hierarchies of the MAGs were inferred using Genome Taxonomy Database Toolkit (GTDB-Tk) (Chaumeil et al. 2020).

### Phylogenetic- and genome-relatedness analyses

RMAR6-6 and MAG 12 belonging to the genera with significantly higher abundances in AS than in BS were analyzed phylogenetically based on their genome sequences. Sequences of 120 bacterial marker proteins encoded by housekeeping genes were extracted from the genomes of RMAR6-6 and MAG 12 and their closely related type strains and aligned using the GTDB-Tk software (Chaumeil et al. 2020). Phylogenetic trees based on the concatenated protein sequences were constructed using the maximum-likelihood algorithm in MEGA7 (Kumar et al. 2016). The ANI values between RMAR6-6 and MAG 12 and their closely related type strains were calculated using the Orthologous Average Nucleotide Identity Tool (OAT) software (Lee et al. 2016).

### Bioinformatic analysis of strains RMAR6-6 and MAG 12 and *P*. *purpureum* CCMP1328

All predicted protein sequences of RMAR6-6 (CP019630) and MAG 12 (CP098402) and *P*. *purpureum* CCMP1328 (VRMN00000000) were downloaded from the GenBank database and functionally annotated using the KEGG Automatic Annotation Server (KASS) (https://www.genome.jp/tools/kaas/) (Moriya et al. 2007) and eggNOG-mapper (http://eggnogmapper.embl.de/) (Huerta-Cepas et al. 2017). In addition, the presence or absence of genes related to metabolic pathways or features was manually curated through BLASTP analysis of reference protein sequences available in the UniProt database (https://www.uniprot.org) against the genomes. Based on the functionally annotated information, the metabolic capabilities of RMAR6-6 and MAG 12 and *P*. *purpureum* CCMP1328 were drawn graphically. The metabolic features of RMAR6-6 and *P*. *purpureum* CCMP1328 derived from the bioinformatic analyses were experimentally evaluated, as described below.

### Production test of vitamins, dimethylsulfoniopropionate (DMSP), phenylacetate (PAA), and utilization test of DMSP

RMAR6-6 was aerobically cultured for 48 h at 25 °C in 5 mL of L1 medium supplemented with 5.5 mmol/L glucose (for vitamin production), L1 medium supplemented with 5.5 mmol/L glucose and 0.5 mmol/L methionine (for DMSP production), and L1 medium supplemented with phenylalanine (for PAA production). *P*. *purpureum* CCMP1328 was cultured for 48 h at 25 °C under the light/dark cycle in 25 mL of L1 medium (for vitamin production test), L1 medium supplemented with 0.5 mmol/L methionine (for DMSP production test), and L1 medium supplemented with phenylalanine (for PAA production test). To assess the utilization of DMSP by RMAR6-6 and *P*. *purpureum* CCMP1328, RMAR6-6 was cultivated for 2 days at 25 °C in 5 mL of L1 medium supplemented with 0.5 mmol/L DMSP, and *P. purpureum* CCMP1328 was cultivated for 4 days at 25 °C under the light/dark cycle in 25 mL of L1 medium supplemented with 0.5 mmol/L DMSP.

### Analysis of vitamins, DMSP, and PAA

Vitamins, DMSP, and PAA in the cultures were analyzed using liquid chromatography quadrupole time-of flight mass spectrometry (LC-Q-TOF–MS). To this end, the cultures of RMAR6-6 and* P*. *purpureum* CCMP1328 were freeze-dried and mixed with 500 μL of methanol/acetonitrile/water solution (2:2:1, v/v). After vigorous mixing with a vortex for 3 min, the mixtures were centrifuged at 18,000×*g* for 3 min and filtered through a 0.2 μm polyvinylidene fluoride filter (BioFact, South Korea). Vitamins, DMSP, and PAA in the filtrate were analyzed using an LC-Q-TOF–MS system comprised a 1290 Infinity UHPLC, an Agilent Poroshell 120 HILIC-Z column (2.1 mm × 100 mm), and a 6550 iFunnel Q-TOF mass spectrometer (Agilent Technologies, USA). The injection volume was 2 μL, and water (solvent A) and acetonitrile (solvent B) containing 5 mmol/L ammonium formic acid were used as mobile phases at a flow rate of 0.4 mL/min with the following gradient: 0 min, 95% B; 0.5 min, 95% B; 3.5 min, 70% B; 4.1 min, 58% B; 4.6 min, 50% B; 6.5 min, 95% B; and 10 min, 95% B. Mass spectrometry was performed under the following conditions: polarity, positive and negative; gas temperature, 250 °C; nebulizer, 35 psi; capillary, ( +) 4000 V; MS range, 50–1500 m/z; MS/MS range, 30–1500 m/z; and collision-induced dissociation energy, 20 eV. Vitamins, DMSP, and PAA were searched for the total LC-Q-TOF–MS data by using the "find by formula" function (Agilent MassHunter Qualitative Analysis B10.0 software) and confirmed using authentic compounds purchased from chemical companies.

### Effect of DMSP and PAA on the growth of axenic *P*. *purpureum* CCMP1328

Axenic cultures of *P*. *purpureum* CCMP1328 cultured in L1 medium for 14 days were inoculated into fresh L1 medium (20% inoculation) containing no addition; 0.5 mmol/L DMSP; 5% NaCl (salt stress); 0.5 mmol/L DMSP + 5% NaCl (salt stress); 0.5 mmol/L H_2_O_2_ (oxidative stress); 0.5 mmol/L DMSP + 0.5 mmol/L H_2_O_2_ (oxidative stress); and 1–10 μmol/L PAA, and the resulting cultures were cultivated for 9 days under the light/dark cycle at 25 °C. The growth of *P*. *purpureum* CCMP1328 cells was assessed based on the fluorescence emission of phycoerythrin in red algae using a BioTek Synergy Mx microplate reader (BioTek, USA) at Ex/Em = 495/580 nm.

### Effect of nitrogen-fixing ability of strain RMAR6-6 on the growth of axenic *P*. *purpureum* CCMP1328

To investigate the nitrogen-fixing ability of RMAR6-6 and its effect on the growth of axenic *P*. *purpureum* CCMP1328, a 2 bp deletion knockout mutant of the *fixH* gene (Fix family protein, B0E33_RS404995) in the putative nitrogen-fixing gene cluster of RMAR6-6 was constructed through single-crossover recombination using the bacterial strain, plasmids, and primer sets listed in Supplementary Table S2, as described previously (Baek et al. 2022). The nitrogen-fixing ability of RMAR6-6 was assessed by comparing the growth of wild-type RMAR6-6 and mutant RMAR6-6∆*fixH* in both L1 medium and nitrate-free L1 medium (L1 medium without the addition of nitrate) supplemented with 5.5 mmol/L glucose at 25 °C for 48 h. For assessing the effect of the nitrogen-fixing ability of RMAR6-6 on the growth of red algae, wildtype RMAR6-6 and mutant RMAR6-6∆*fixH* were cultured in L1 medium with 5.5 mmol/L glucose at 25 °C, and axenic* P*. *purpureum* CCMP1328 was cultured in L1 medium under the light/dark cycle at 25 °C. Cells of wild-type RMAR6-6 and mutant RMAR6-6∆*fixH* were inoculated into both L1 and nitrate-free L1 media containing approximately 10^3^ *P*. *purpureum* cells/mL to a final concentration of approximately 10^5^ bacterial cells/mL, and incubated at 25 °C under the light/dark cycle for 35 days. The growth of *P*. *purpureum* was assessed by measuring fluorescence (see the previous section).

### Genome-wide transcriptome analysis of strain RMAR6-6 and *P*. *purpureum* CCMP1328

For the transcriptome analysis of *P*. *purpureum* CCMP1328 and RMAR6-6 in monoculture, axenic *P*. *purpureum* CCMP1328 was cultured (in triplicate) in 175 cm^2^ cell culture flasks containing 100 mL of L1 medium for 14 days at 25 °C under the light/dark cycle, and RMAR6-6 was aerobically cultured (in triplicate) in 175 cm^2^ cell culture flasks containing 100 mL of L1 medium supplemented with 5.5 mmol/L glucose for 24 h at 25 °C. In addition, for the transcriptome analysis of *P*. *purpureum* CCMP1328 and RMAR6-6 in co-culture, RMAR6-6 (10^5^ cells/mL) and *P*. *purpureum* CCMP1328 (10^3^ cells/mL) were inoculated together into 100 mL of L1 medium and co-cultured for 14 days at 25 °C under the light/dark cycle. The mono- and co-cultured cells were harvested rapidly by centrifugation (32,310×*g*, 5 min, 4 °C) and the triplicated harvested cells were combined together. TRIzol reagent (Thermo Fisher Scientific, USA) was used for the extraction of total RNAs from the monoculture of *P*. *purpureum* CCMP1328 and the co-culture of *P*. *purpureum* CCMP1328 and RMAR6-6, whereas RNeasy Mini Kit (Qiagen, USA) was used for the extraction of total RNAs from the monoculture of RMAR6-6, according to the manufacturers’ instructions. The extracted RNAs were treated with DNase I (Takara, Japan), and algal and bacterial rRNA was depleted using the TruSeq Stranded Total RNA with Ribo-Zero Plant (Illumina, USA) and Illumina Stranded Total RNA with Ribo-Zero Plus kits, respectively. The purified mRNA was sequenced using an Illumina HiSeq X platform (× 151 bp) at Macrogen and the resulting sequencing reads were trimmed (Q < 30), and reads shorter than 50 nucleotides were removed using Sickle. High-quality sequencing reads derived from the mono- and co-cultured cells of *P*. *purpureum* CCMP1328 and RMAR6-6 were mapped to coding sequences (CDS) of the genomes of RMAR6-6 and *P*. *purpureum* CCMP1328 using Burrows Wheeler Aligner software (Li and Durbin 2009) with the default option. Read counts mapped to CDS were calculated using SAMtools (http://samtools.sourceforge.net) and BEDTools (https://bedtools.readthedocs.io/en/latest/) and normalized by RPKM. Differential gene expressions between mono- and co-culture of RMAR6-6 and *P*. *purpureum* CCMP1328 were indicated using fold change of RPKM values.

## Results

### Phylogeny of marine red algae and bacterial communities

Nine xenic marine red algal cultures (*Asparagopsis* (*As*.) *taxiformis*, *Grinnellia* sp., *Rhodymenia palmata*, *Dixoniella grisea*, *Porphyridium purpureum*, *Chroodactylon ornatum*, *Rhodosorus* (*Rhodo*.) *marinus*, *Stylonema alsidii*, and *Stylonema cornu-cervil*) were confirmed taxonomically using their ribulose-1,5-bisphosphate carboxylase gene (*rbcL*) sequences (Supplementary Table S1), which showed that they were widely distributed in the *Rhodophyta* (Supplementary Fig. S1). After subculturing them for more than six months under the same conditions, bacterial communities in BS and AS of algae were analyzed at the phylum and genus levels. At the phylum level, *Bacteroidota* and *Pseudomonadota* predominated in seawater and all red algae (Supplementary Fig. S2), but *Pseudomonadota* was more abundantly identified in AS than in BS as well as seawater, suggesting a close association between the growth of *Pseudomonadota* with marine red algae. *Planctomycetota*, *Bacillota*, *Actinomycetota*, and *Hydrogendentes* that were not identified in seawater were identified in AS, although they were minor, suggesting that their growth may also be closely associated with marine red algae. In particular, *Planctomycetota* was identified only in AS, indicating presumably a robust symbiotic association with marine red algae.

**Fig. 1:**
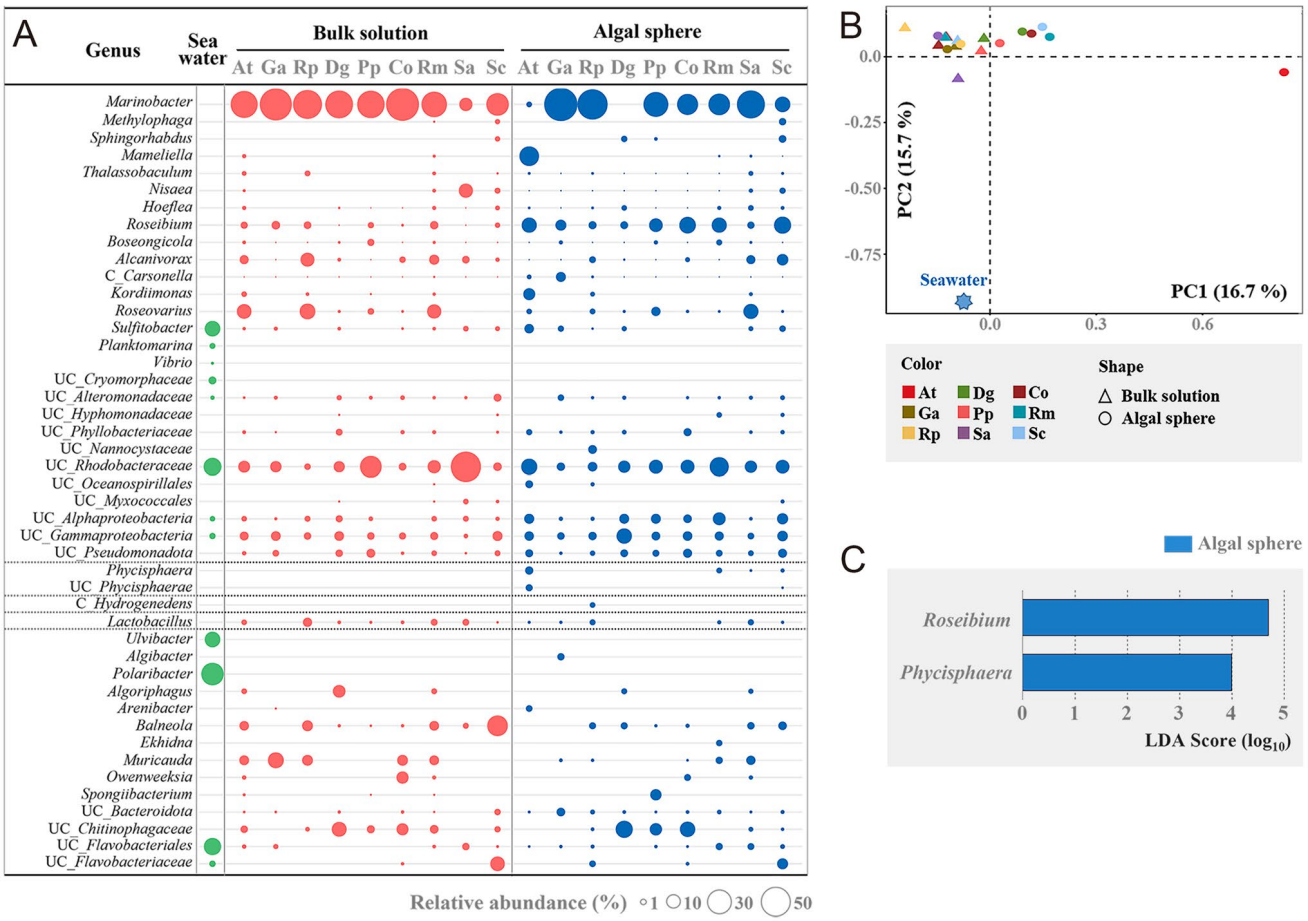
Bacterial communities at the genus level in the bulk solution and algal sphere of marine red algae (**A**) and their principal component analysis (**B**). LEfSe (linear discriminant analysis effective size) analysis showing bacterial groups with significantly different abundances between the bulk solution and algal sphere of marine red algae (**C**). “Others” represents the sum of bacterial genera with less than 1% relative abundances in all samples. UC, unclassified; At, *Asparagopsis taxiformis*; Ga, *Grinnellia americana*; Rp, *Rhodymenia palmata*; Dg, *Dixoniella grisea*; Pp, *Porphyridium purpureum*; Co, *Chroodactylon ornatum*; Rm, *Rhodosorus marinus*; Sa, *Stylonema alsidii*; Sc, *Stylonema cornu-cervil*. Bacterial community of seawater sampled from the East Sea of South Korea was used as a reference

At the genus level, *Polaribacter*, *Sulfitobacter*, *Ulvibacter*, *Planktomarina*, *Vibrio* and some other unclassified bacterial groups were identified abundantly in seawater, but most of them were not common in BS as well as in AS (Fig. 1A), suggesting that their growth may not be greatly dependent on marine red algae. However, *Marinobacter*, *Roseibium*, *Roseovarius*, *Balneola*, *Alcanivorax* and *Muricauda* that were not identified commonly in seawater were abundant in BS and AS (Fig. 1A), suggesting that they may be microbiota closely associated with marine red algae. *Marinobacter* was the most abundant genus in the majority of marine red algae, and *Roseibium* was commonly associated with all marine red algae regardless of AS and BS. Interestingly, *Phycisphaera* belonging to the phylum *Planctomycetota* was identified in some AS but not BS.

Bacterial communities in BS and AS of marine red algae were relatively similar, except for *As. taxiformis*, but they were clearly distinct from those in seawater (Fig. 1B). This indicates that they are greatly affected by marine red algae in BS and AS in laboratory cultures due to their closed system nature, unlike the open system of seawater. Algal microbial communities are strongly influenced by the metabolic characteristics of the algae. Therefore, the relationships between the phylogeny of marine red algae and the bacterial communities in their BS and AS were analyzed, but no specific relationship was identified in both BS and AS (Supplementary Fig. S3). To identify microbiota more tightly associated with marine red algae, rather than just using nutrients derived from marine red algae, the LEfSe analysis between the bacterial communities of AS and BS was performed. Thus, *Roseibium* and *Phycisphaera* were shown to be significantly more abundant in AS than in BS (Fig. 1C).

### Isolation and genome sequencing of strain RMAR6-6

Isolation of bacterial strains belonging to the genera *Roseibium* and *Phycisphaera* from algal cultures was attempted. Bacterial strains belonging to the genera, abundantly identified in AS were mostly isolated, and a putative *Roseibium* strain (designated RMAR6-6) was also recovered successfully. RMAR6-6 was most closely related to *Roseibium aggregatum* IAM 12614^T^, with a 100% 16S rRNA gene sequence similarity, suggesting that RMAR6-6 is a member of the genus *Roseibium*. The genome of *Roseibium* RMAR6-6 consisted of one circular chromosome (6149 kb) and two circular plasmids (407 kb and 94 kb) with a 59.4% G + C content and 6163 genes (Supplementary Fig. S4). However, despite various attempts, the isolation of bacterial strains corresponding to *Phycisphaera* failed, suggesting that their growth may require a strict symbiotic relationship with marine red algae. Bacterial members classified as *Phycisphaera* in the bacterial community analysis of AS samples only shared less than 83% 16S rRNA gene sequence similarities with the only cultured *Phycisphaera* species (*Phycisphaera mikurensis*), suggesting that they may be a new genus member that has not yet been cultured, not *Phycisphaera*.

### Shotgun metagenome sequencing and acquisition of MAG 12

A total of 4.74 Gb of metagenome sequencing reads were generated from the xenic culture of *Rhodo*. *marinus*. The assembly of the metagenome sequencing reads yielded 48,335 contigs (> 100 bp), amounting to 145.8 Mb in total size. After metagenomic binning of the contigs, 17 dereplicated metagenome-assembled genomes (MAGs) with relatively high completeness and low contamination rates were generated (Supplementary Table S3). Among 17 MAGs, two MAGs (MAGs 12 and 13) were classified as members of the phylum *Planctomycetota* that were identified only in AS of marine red algae. However, the taxonomy showed that MAG 12, classified into *Planctomycetota*; *Phycisphaerales*; and *Phycisphaerae*, corresponded to *Phycisphaera* that was more commonly identified in AS than in BS. MAG 12 consisted of a single contig of 3.62 Mb, the largest contig in the metagenomic assembly, with a 66.2% G + C content and 3120 genes.

### Phylogenetic positions of strains RMAR6-6 and MAG 12

**Fig. 2:**
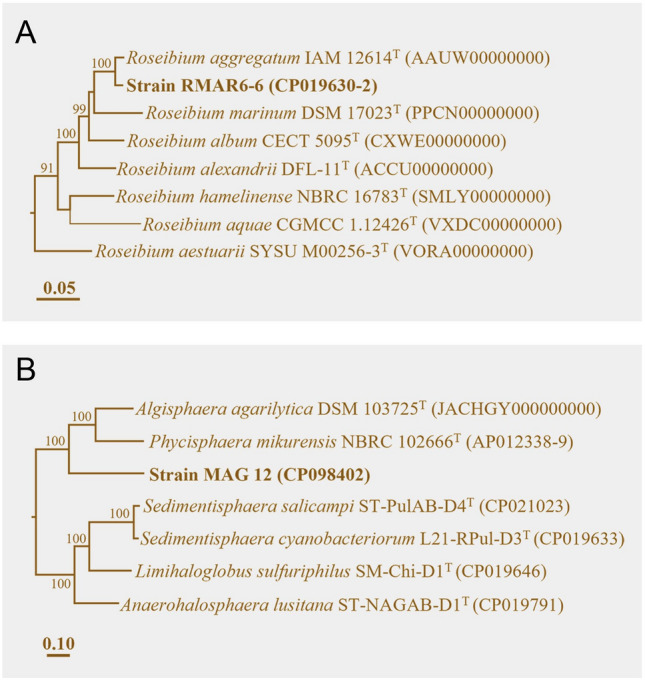
Phylogenetic trees showing the phylogenetic positions of *Roseibium* sp. RMAR6-6 (**A**) and strain MAG 12 (**B**), based on the concatenated 120 bacterial marker protein sequences. Bootstrap values for 1000 replicate analyses (> 70%) are shown at branching points. Scale bars represent the changes per amino acid position

Genome sequence-based phylogenetic analysis showed that RMAR6-6 was clustered tightly with *R*. *aggregatum* IAM 12614^T^ within the genus *Roseibium* (Fig. 2A). The Average nucleotide identity (ANI) value between RMAR6-6 and *R*. *aggregatum* IAM 12614^T^ was 87.5%. This was lower than the criterium for the prokaryotic species delineation threshold (ANI, ca. 95%) (Chun et al. 2018), suggesting that RMAR6-6 is a novel species of the genus *Roseibium*, although the 16S rRNA gene sequence similarity between RMAR6-6 and *R*. *aggregatum* IAM 12614^T^ was 100%. MAG 12 formed a distant phyletic lineage from *Algisphaera agarilytica* DSM 103725^T^ and *Phycisphaera mikurensis* NBRC 102666^T^ within the family *Phycisphaeraceae* (Fig. 2B) confirming that MAG 12 is not a *Phycisphaera* species. ANI values between MAG 12 and other cultured bacterial type strains were less than 67.0%.

*Porphyridium purpureum* CCMP1328, which is currently available as an axenic culture and has been genome-sequenced (Bhattacharya et al. 2013; Lee et al. 2019), was selected as a model red marine alga for further interaction studies. To gain insights into metabolic relationships between symbiotic bacteria and marine red algae, the metabolic features of *Roseibium* RMAR6-6, MAG 12, and *P*. *purpureum* CCMP1328 were investigated through bioinformatic analysis.

### Metabolic features of strain RMAR6-6 and its symbiotic relationships with *P*. *purpureum*

**Fig. 3:**
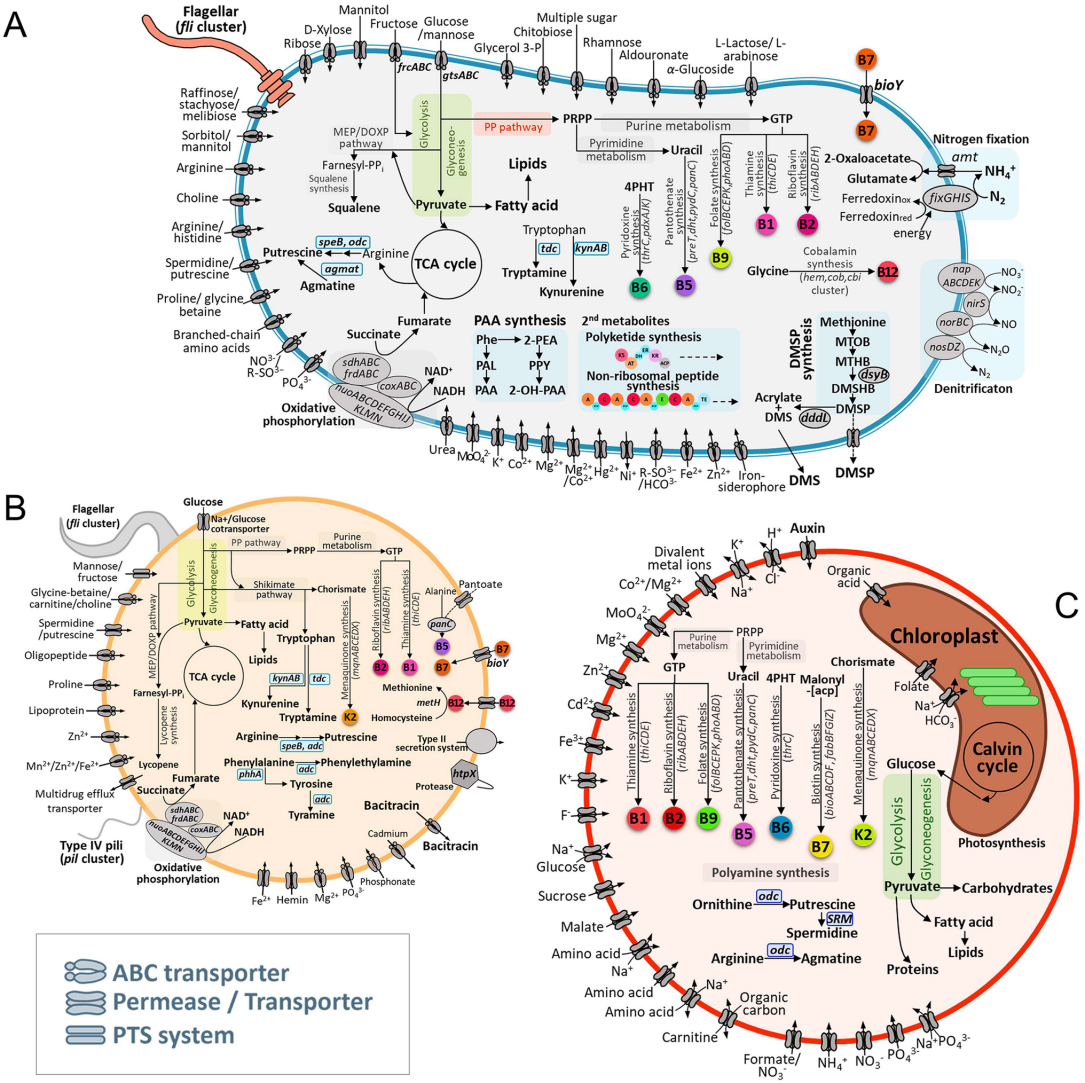
Metabolic features of *Roseibium* RMAR6-6 (**A**) and strain MAG 12 (**B**) and a marine red alga *Porphyridium purpureum* CCMP1328 (**C**) inferred by the bioinformatic analysis. *adc*, arginine decarboxylase; *agmat,* agmatinase; *amt*, ammonium transporter; *cbi* and *cob*, cobalamin biosynthesis genes, uroporphyrinogen-III to cobyrinate a,c-diamide; *coxABC*, iron:rusticyanin reductase; *dddL*, dimethylsulfoniopropionate lyase; *dht*, dihydropyrimidinase; *dysB*, methyltransferase; *folBCEPK*, tetrahydrofolate biosynthesis genes; *fixGHIS*, nitrogen fixation protein; *frdABC*, fumarate reductase; *hem*, glycine to uroporphyrinogen-III synthase; *htpX*, polypeptide protease; *kynAB*, kynurenine formamidase and indoleamine 2,3-dioxygenase; *panC*, pantothenate synthetase; *pdxAJK*, pyridoxine biosynthesis genes; *phhA*, phenylalanine-4-hydroxylase; *phoABD*, alkaline phosphatse; *preT*, dihydropyrimidine dehydrogenase; *metH*, cobalamin-dependent methionine synthase; *napABCEDK*, nitrate reductase; *nirS*, nitrite reductase; *norBC*, respiratory nitric oxide reductase; *nosDZ*, nitrous oxide reductase; *nuoABCDEFGHIJKLMN*, NADH:quinone oxidoreductase; *odc*, ornithine decarboxylase; *ribABDEH*, riboflavin biosynthesis genes; *sdhACB*, succinate dehydrogenase; *speB*, arginine decarboxylase; *tdc*, tyrosine decarboxylase; *thiCDE*, thiamin biosynthesis genes; *thrC*, threonine synthase; DMS, dimethylsulfide; DMSHB, 4-dimethylsulfonio-2-hydroxy-butyrate; DMSP, dimethylsulfoniopropionate; PAA, phenylacetic acid; PAL, phenylacetaldehyde; PAPS, phosphoadenosine 5′-phosphosulfate; PEA, 2-phenylethanol; Phe, phenylalanine; PP pathway, pentose phosphate pathway; 4-PHT, 4-phosphohydroxy-l-threonine; PRPP, phosphoribosylpyrophosphate; PPY, phenylpyruvate; MEP/DOXP, 2-C-methyl-d-erythritol 4-phosphate/1-deoxy-d-xylulose 5-phosphate; MTOB, 4-methylthio-2-oxobutanoate; MTHB, 4-methylthio-2-hydroxybutyrate; TCA, tricarboxylic acid cycle

RMAR6-6 harbors various uptake transport systems for diverse compounds and minerals, including free sugars, sugar alcohols, amines, amino acids, and metals (Fig. 3A), as well as complete glycolysis, gluconeogenesis, and pentose phosphate pathways, a complete tricarboxylic acid (TCA) cycle, and an oxidative phosphorylation system, representing aerobic respiration. Also, RMAR6-6 harbors a complete denitrification system capable of reducing nitrate to nitrogen gas, suggesting that this strain is a facultative aerobic bacterium capable of utilizing nitrate as an electron acceptor. The versatile metabolic capability of RMAR6-6 may well be a key characteristic of *Roseibium* RMAR6-6 as a common microbiota that can survive with a variety of marine red algae.

RMAR6-6 harbors complete gene sets for the synthesis of numerous B vitamins, including thiamine (B1), riboflavin (B2), pantothenic acid (B5), pyridoxine (B6), folic acid (B9) and cobalamin (B12), possibly required by the auxotrophic hosts, such as marine algae (Cooper et al. 2019); among them, the production of riboflavin and pyridoxine was confirmed by LC-Q-TOFMS analysis in this study (Supplementary Fig. S5). RMAR6-6 harbors a gene encoding biotin (B7) transporter (*bioY*) that likely serves to import biotin produced by the partner (Guillén-Navarro et al. 2005). RMAR6-6 possesses a gene (B0E33_RS08010) encoding bacterioferritin as a siderophore. Additionally, it harbors genes capable of converting arginine (*speB* and *odc*) and agmatine (*agmat*) to putrescine (Fig. 3A). The bioinformatic analysis revealed that RMAR6-6 harbors type 1 polyketide synthase (PKS, locus_tag: B0E33_RS27900–10) and nonribosomal peptide synthase (NRPS, locus_tag: B0E33_RS28245) genes in the chromosome and an incomplete NRPS operon (B0E33_RS28880–910) in plasmid 1. The polyketides or NRPS products synthesized by RMAR6-6 could function as competitive weapons against other algae-associated or harmful microbes. This characteristic of RMAR6-6 may well be a crucial factor in influencing the composition of a healthy microbial community in the AS.

**Fig. 4:**
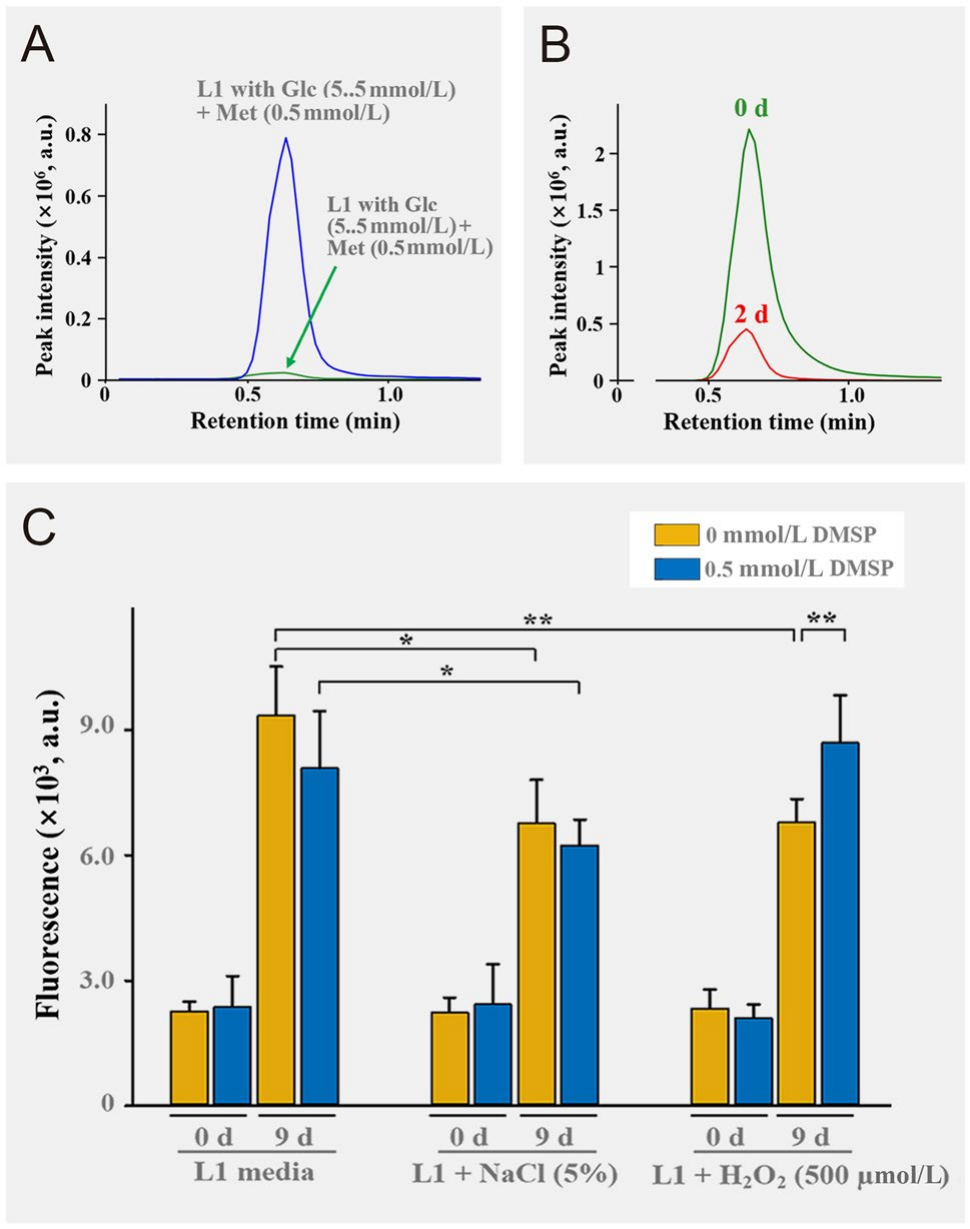
Production of dimethylsulfoniopropionate (DMSP) from methionine by *Roseibium* RMAR6-6 (**A**). The DMSP productions were tested in L1 media supplemented with glucose (Glc, 5.5 mmol/L), and with Glc (5.5 mmol/L) and methione (Met, 0.5 mmol/L). Utilization of DMSP by *Roseibium* RMAR6-6 in L1 media supplemented with Glc (5.5 mmol/L) and DMSP (0.5 mmol/L) (**B**). DMSP was analyzed using LC-Q-TOF–MS on day 2 for DMSP production and days 0 and 2 for DMSP utilization. Effects of DMSP on the growth of axenic *Porphyridium purpureum* CCMP1328 under salt and oxidative stress conditions (**C**). The analyses were performed in triplicate, and the error bars represent standard deviations. a.u., arbitrary unit. *p*-values < 0.05 (*) or < 0.01 (**)

The genomic analysis showed that RMAR6-6 harbors *dsyB* and *dddL* genes encoding a methyltransferase-like protein (B0E33_RS19275) and dimethylsulfoniopropionate (DMSP) lyase (B0E33_RS20200), involved in the synthesis and utilization of DMSP, respectively (Curson et al. 2017, 2018). LC-Q-TOF–MS analysis showed clearly that RMAR6-6 also has abilities to produce DMSP from methionine (Fig. 4A) and to metabolize DMSP (Fig. 4B). However, no gene involved in the synthesis of DMSP was identified in the genome of *P*. *purpureum* CCMP1328, indicating that the red alga *P*. *purpureum* cannot synthesize DMSP. To investigate the effects of DMSP produced by RMAR6-6 on the growth of marine red algae, 0.5 mmol/L DMSP was added to the cultures of axenic *P*. *purpureum* CCMP1328 under salt and oxidative stresses (Fig. 4C). Although DMSP could not protect axenic *P*. *purpureum* culture from salt stress, it did protect axenic *P*. *purpureum* culture from oxidative stress. These results suggest that DMSP produced by *Roseibium* RMAR6-6 could protect marine red algae from oxidative stress caused by photosynthesis.

**Fig. 5:**
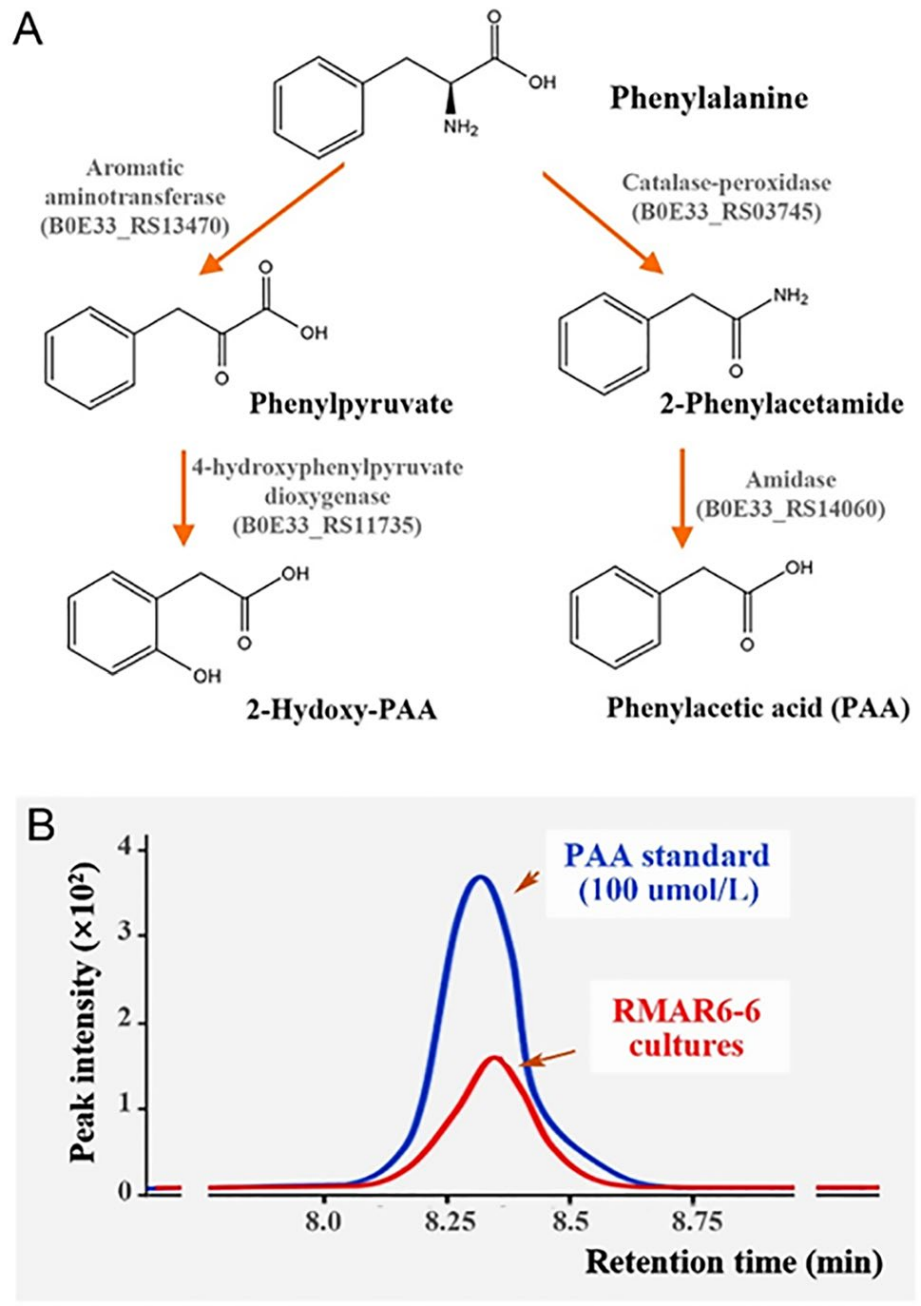
Predicted synthesis pathway of phenylacetate (PAA) and 2-hydroxy-PAA (**A**) and LC-Q-TOF-MS ion chromatograms showing the production of PAA by *Roseibium* RMAR6-6 in L1 media supplemented with glucose (**B**)

The genomic analysis showed that RMAR6-6 harbors genes involved in the synthesis of phenylacetate (PAA) and 2-hydroxy-PAA (Fig. 5A). In the analysis of the metabolites, PAA was detected at very low concentrations in the culture suspension of RMAR6-6 (Fig. 5B); 2-hydroxy-PAA was not detected in this study (data not shown). To investigate the effects of PAA produced by RMAR6-6 on the growth of marine red algae, various concentrations of PAA were added to the cultures of axenic *P*. *purpureum* CCMP1328, but no distinct effect of PAA on the growth of axenic *P*. *purpureum* was observed (data not shown). However, because PAA is purported to have a growth regulating effect on both marine red algae and brown algae (Fries 1977), PAA or 2-hydroxy-PAA produced by RMAR6-6 may contribute to the growth promotion or stress tolerance improvement of marine algae in marine environments.

**Fig. 6:**
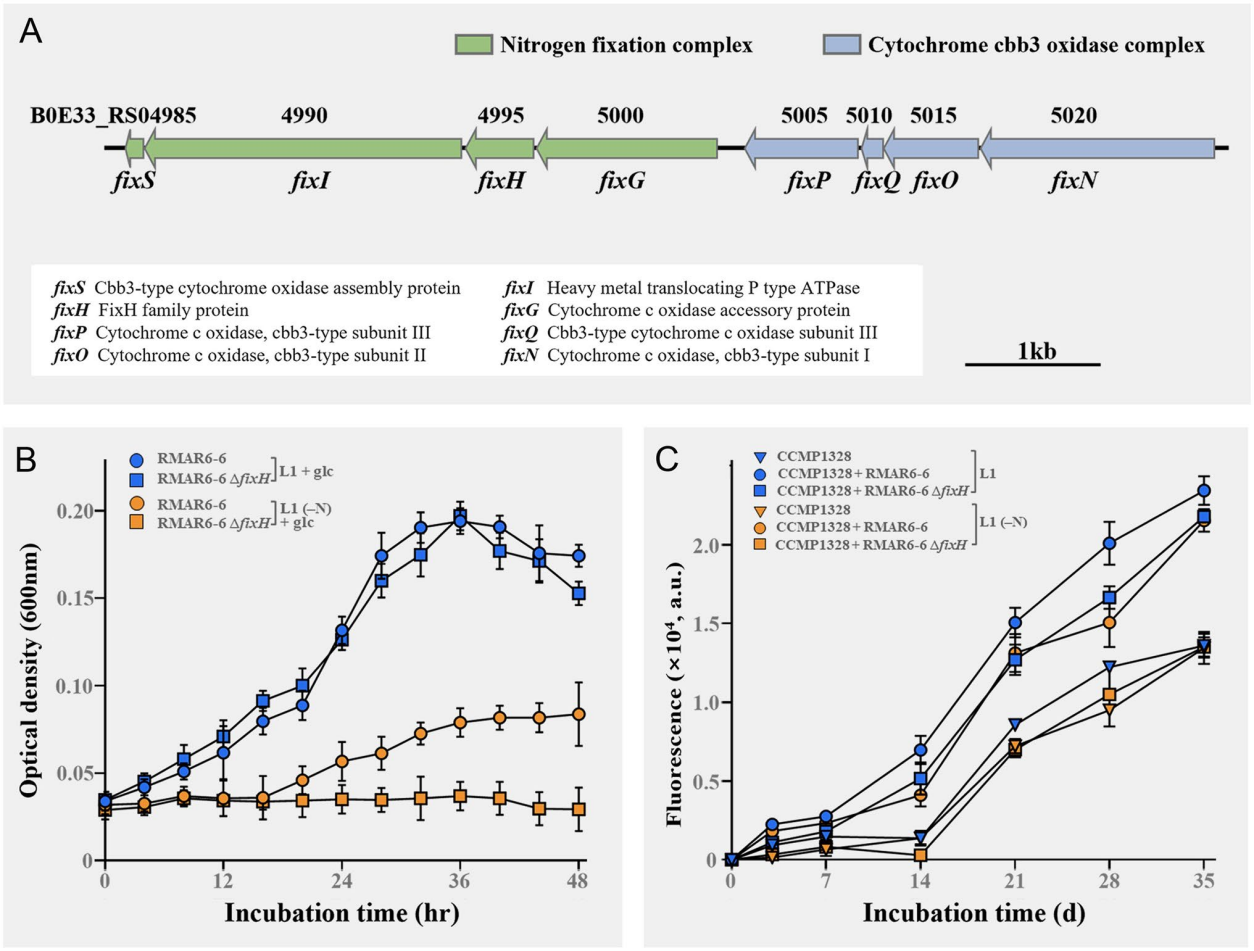
Organization and predicted functions of genes in the putative nitrogen-fixing gene cluster of *Roseibium* RMAR6-6 (**A**). Growth of wild-type RMAR6-6 and mutant RMAR6-6 ∆*fixH* in L1 medium with/without nitrate supplemented with glucose (**B**), and the effects of wild-type and mutant strains on the growth of axenic *Porphyridium purpureum* CCMP1328 in L1 medium with/without nitrate (**C**). L1 (–N) represents L1 medium without the addition of nitrate. glc, glucose; a.u., arbitrary unit

The genomic analysis showed that RMAR6-6 harbors a putative nitrogen fixation gene cluster (*fixNOQPGHIS*) (Fig. 5A), similar to that in rhizobia (Delgado et al. 1998); thus, its nitrogen-fixing ability was assessed through growth tests. The wild-type RMAR6-6 and its *fixH* gene knockout mutant RMAR6-6 ∆*fixH* grew similarly in L1 medium containing nitrate (Fig. 6B). However, the growth of mutant RMAR6-6 ∆*fixH* was not observed in nitrate-free L1 medium, whereas wild-type RMAR6-6 grew relatively well, even in nitrate-free L1 medium. This suggests that RMAR6-6 has the ability to fix nitrogen through the nitrogen fixation gene cluster. In addition, the effects of wild-type RMAR6-6 and mutant RMAR6-6 ∆*fixH* on the growth of *P*. *purpureum* CCMP1328 were investigated (Fig. 6C). In nitrate-free L1 medium, wild-type RMAR6-6 clearly enhanced the growth of *P*. *purpureum* CCMP1328, whereas mutant RMAR6-6 ∆*fixH* did not. This suggests that the nitrogen-fixing ability of *Roseibium* RMAR6-6 enhances the growth of *P*. *purpureum*. However, the axenic culture of *P*. *purpureum* CCMP1328 without RMAR6-6 showed only a slightly better growth in L1 medium containing nitrate than in nitrate-free L1 medium. This suggests that there may be other limiting factors necessary for the growth of *P*. *purpureum* CCMP1328, besides a nitrogen source in nitrate-free L1 medium. Accordingly, in L1 medium containing nitrate, not only wild-type RMAR6-6 but also mutant RMAR6-6 ∆*fixH* clearly enhanced the growth of *P*. *purpureum* CCMP1328. This showed that besides nitrogen source, other factors, such as vitamins, bacterioferritin, polyamines, DMSP and PAA, produced by RMAR6-6 may well enhance the growth of *P. purpureum* CCMP1328.

### Metabolic features of strain MAG 12

MAG 12, which was identified only in AS (Fig. 1A), also harbors complete glycolysis, gluconeogenesis and pentose phosphate pathways, a complete TCA cycle and an oxidative phosphorylation system, representing aerobic respiration, like RMAR6-6 (Fig. 3B). However, MAG 12 has small numbers of transport systems that could utilize only limited carbon compounds and minerals compared to RMAR6-6. Also, MAG 12 harbors limited genes involved in the synthesis of vitamins B1, B2 and B5 relative to strain RMAR6-6. Instead, MAG 12 has vitamin B7 and B12 transporters to import these vitamins produced by another symbiotic partner. MAG 12 does not harbor genes for DMSP, PAA and iron siderophore syntheses and nitrogen fixation that may benefit the growth of marine algae. RMAR6-6 harbors a complete gene set for squalene biosynthesis, whereas MAG 12 harbors a complete gene set for lycopene biosynthesis.

### Metabolic features of *P. purpureum* CCMP1328

A marine red alga *P. purpureum* CCMP1328 harbors complete glycolysis and gluconeogenesis pathways, a complete TCA cycle and a complete Calvin cycle, representing a photoautotrophic organism (Fig. 3C). Also, it harbors genes responsible for the synthesis of various B vitamins, including B1, B2, B5, B6, B7 and B9, but not vitamin B12, which is required for DNA synthesis, fatty acid and amino acid metabolism and photosynthesis. Therefore, the cobalamin-producing ability of *Roseibium* RMAR6-6 may be a key characteristic for enhancing the growth of *P*. *purpureum* CCMP1328 (Fig. 6C) (Cooper et al. 2019; Kazamia et al. 2012). However, *P*. *purpureum* CCMP1328 harbors genes for biotin biosynthesis, which two symbiotic bacteria do not harbor, indicating that the biotin-producing ability of *P*. *purpureum* CCMP1328 may be an important metabolic feature that supports the growth of heterotrophic bacteria, such as RMAR6-6 and MAG 12, besides representing a carbon source.

It has been reported that DMSP can be also metabolized by many marine algae, such as *Emiliania huxley* and *Symbiodinium*, besides bacteria in the ocean (Amin et al. 2015; Matthews et al. 2020). However, neither DMSP-producing genes nor DMSP-catabolic genes were identified in the genome of *P*. *purpureum* CCMP1328. Also, the genes for PAA synthesis or metabolism were not identified in *P*. *purpureum* CCMP1328. The absence of DMSP and PAA-metabolic genes in *P*. *purpureum* CCMP1328 was confirmed by LC-Q-TOF–MS analysis (data not shown). Therefore, DMSP and PAA produced by *Roseibium* RMAR6-6 may provide beneficial effects to enhance the growth or survival of marine red algae in the ocean.

### Transcriptomic analysis of strain RMAR6-6 and *P. purpureum* CCMP1328 in mono- and co-culture

**Fig. 7:**
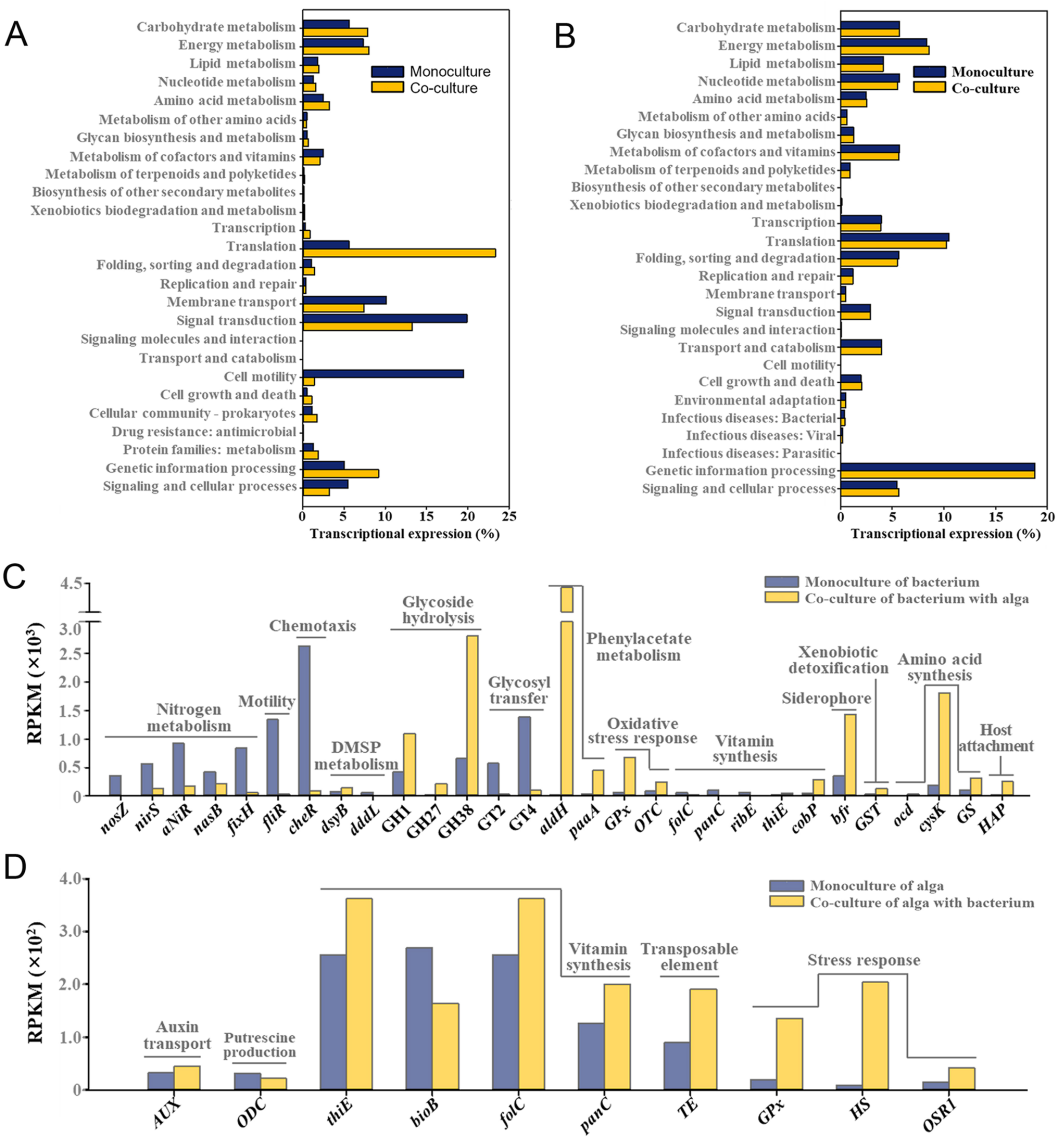
Transcriptional expressions at the secondary level of KEGG functional categories of *Roseibium* RMAR6-6 (**A**) and *Porphyridium purpureum* CCMP1328 (**B**) in mono- and co-culture. Representative genes of strains RMAR6-6 (**C**) and CCMP1328 (**D**) showing differential transcriptional expressions (RPKM, reads per kilobase of each coding sequence per million mapped reads) between mono- and co-culture. *nosZ*, nitrous oxide reductase; *nirS*, nitrite reductase; *aNiR*, assimilatory nitrate reductase; *fixH*, nitrogen fixation protein; *fliR*, flagellar biosynthesis protein; *cheR*, chemotaxis protein methyltransferase; *dsyB*, methylthiohydroxybutyrate methyltransferase; *dddL*, dimethylsulfoniopropionate lyase; GH1, glycoside hydrolase family 1; GH27, glycoside hydrolase family 27; GH38, glycoside hydrolase family 38; GT2, glycosyltransferase family 2; GT4, glycosyltransferase family 4; *aldH*, aldehyde dehydrogenase; *paaA*, phenylacetic acid degradation protein; *GPx*, glutathione peroxidase; *OTC*, ornithine carbamoyltransferase; *folC*, dihydrofolate synthase/folylpolyglutamate synthase; *panC*, pantothenate synthetase; *ribE*, riboflavin synthase; *thiE*, thiamine-phosphate synthase; *cobP*, bifunctional adenosylcobalamin biosynthesis protein; *bfr*, bacterioferritin; *GST*, glutathione S-transferase; *ocd*, ornithine cyclodeaminase; *cysK*, cysteine synthase A; *GS*, glutamate synthase; *HAP*, host attachment protein; *AUX*, auxin transporter; *ODC*, ornithine decarboxylase; *bioB*, biotin synthase; TE, transposable elements; HS, heat shock proteins; *OSR1*, serine/threonine-protein kinase

To investigate metabolic relationships between RMAR6-6 and *P*. *purpureum* CCMP1328, their transcriptomes in mono- and co-culture were analyzed. Approximately 60.0 to 197.8 million sequencing reads were obtained from mono- and co-cultured cells of RMAR6-6 and *P*. *purpureum* CCMP1328, and only high-quality mRNA sequencing reads after removing low-quality reads were used for transcriptomic analyses (Supplementary Table S5). Scatter plotting comparing the genome-wide differential expressional profiles (RPKM values; reads per kilobase of CDS per million mapped reads values) between mono- and co-culture of strain RMAR6-6 and *P*. *purpureum* CCMP1328 were generated (Supplementary Fig. S6). RMAR6-6 was more differentially expressed between mono- and co-culture than *P*. *purpureum* CCMP1328, perhaps because, in addition to the interaction of RMAR6-6 with CCMP1328, growth conditions (e.g., carbon source, growth rate) were too different between mono- and co-culture of RMAR6-6. Mono- and coculture conditions of *P*. *purpureum* CCMP1328 were almost similar except for the growth of strain RMAR6-6. To further investigate genes involved in the responses or interactions between RMAR6-6 and *P*. *purpureum* CCMP1328, gene expressions of RMAR6-6 and *P*. *purpureum* CCMP1328 in mono- and co-culture were compared based on the KEGG category of transcriptional sequencing reads (Fig. 7). Also, the KEGG-based analysis showed that more KEGG categories of RMAR6-6 were differentially expressed between mono- and co-culture than those of *P*. *purpureum* CCMP1328 (Fig. 7A, B); the transcriptional expression of KEGG categories between mono- and co-culture of *P*. *purpureum* CCMP1328 was almost similar.

In the co-culture of RMAR6-6 with CCMP1328, there was upregulation of genes involved in PAA metabolism (*aldH* and *paaA*), glycoside hydrolysis (GH1, GH27, and GH38), oxidative stress response (*GPx* and *OTC*), siderophore (*bfr*), amino acid synthesis (*cysK* and *GS*), host attachment (*HAP*), xenobiotic detoxification (*GST*) and cobalamin (*cobP*) and DMSP (*dsyB*) syntheses and downregulation of genes involved in nitrogen metabolism (*nosZ*, *nirS*, *aNiR*, *nasB*, and *fixH*), motility (*fliR*), chemotaxis (*cheR*) and glycosyl transfer (GT2 and GT4) compared with the monoculture of RMAR6-6 (Fig. 7C). The genes of RMAR6-6 could potentially be differentially expressed between mono- and co-culture due to distinct culture conditions. Nevertheless, it is plausible that the observed differential expression of these genes is influenced by the co-culture with *P. purpureum* CCMP1328. In particular, the upregulation of PAA metabolism and bacterioferritin, cobalamin, and DMSP syntheses in RMAR6-6 were induced by co-culture with *P*. *purpureum* CCMP1328, which may well contribute to the growth promotion or survival of *P*. *purpureum* CCMP1328 (Cooper et al. 2019; Croft et al. 2005; Fries 1977; Rambo et al. 2020; Strom et al. 2003; Sunda et al. 2002). In addition, in the co-culture of RMAR6-6 with CCMP1328, the upregulation of host attachment and the downregulation of motility and chemotaxis may well be caused by the colonization of RMAR6-6 on the cell surface of *P*. *purpureum* CCMP1328 (Samo et al. 2018; Shibl et al. 2020). The upregulation of oxidative stress response, glycoside hydrolysis and xenobiotic detoxification genes of RMAR6-6 may well be attributed to light irradiation, carbohydrates, and secondary metabolites (the latter two are produced by *P*. *purpureum* CCMP1328) in the co-culture with *P*. *purpureum* CCMP1328, respectively. However, the downregulation of nitrogen metabolism and glycosyl transfer and the upregulation of amino acid synthesis in RMAR6-6 may be attributed to the different growth conditions (carbon source and growth rate) in mono- and co-culture, not to metabolic interactions by the co-culture of RMAR6-6 with *P*. *purpureum* CCMP1328.

The transcriptional profiles of *P*. *purpureum* CCMP1328 in mono- and co-culture were relatively similar. Some genes of *P*. *purpureum* CCMP1328 were differentially expressed in co-culture with RMAR6-6 (Fig. 7D). Genes involved in biotin synthesis (*bioB*) were downregulated, but those involved in the synthesis of thiamine (*bioB*), folate (*folC*) and pantothenic acid (*panC*), stress response (*GPx*, *HS*, and *OSR1*) and putrescine production (*ODC*) were upregulated.

## Discussion

In marine ecosystems, symbiotic interactions between marine phototrophs and heterotrophic bacteria are mediated through a series of metabolic exchanges that may affect the growth or survival of partner organisms. Many heterotrophic bacteria with important roles in the symbiotic relationships with partner marine phototrophs have been suggested (Amin et al. 2015; Rooney-Varga et al. 2005; Seyedsayamdost et al. 2011). For example, *Labrenzia* (presently *Roseibium*) members have been suggested as crucial microbiota potentially engaged in metabolic associations with *Symbiodinium*, a dinoflagellate (Lawson et al. 2018; Maire et al. 2021). However, the precise metabolic interactions between *Roseibium* and marine phototrophs, including *Symbiodinium*, remain unclear. Furthermore, *Phycisphaeraceae* members of the phylum *Planctomycetota* containing *Phycisphaera* have been suggested as an important microbiota closely associated with diverse marine phototrophs, including cyanobacteria, such as *Synechococcus*, dinoflagellates, such as *Gambierdiscus*, and macroalgae (Bondoso et al. 2017; Lage and Bondoso 2014; Rambo et al. 2020; Zheng et al. 2020). However, their metabolic interactions with marine phototrophs were inferred through metagenomic or metaproteomic studies due to their unculturability (Rambo et al. 2020; Zheng et al. 2020).

In this study, we analyzed bacterial communities of nine marine red algal cultures and *Roseibium* and *Phycisphaera* members were identified as potential algae-associated bacteria that can symbiotically interact with marine red algae (Fig. 1). In particular, *Roseibium* members were identified in all nine marine red algal cultures, suggesting that they may be an algae-associated common microbiota that can importantly affect the survival or growth of marine algae. However, because *Roseibium* members were identified commonly in the BS as well as in the AS, their growth may not be highly dependent upon marine red algae. Conversely, *Phycisphaera* members were identified in only four AS and not at all in BS, suggesting that their growth may be highly dependent upon marine algae but not essential for the growth of marine algae. These findings imply that *Roseibium* exhibits a generalist nature, thriving in diverse marine environments and establishing symbiotic associations with various marine algae through a range of interactions crucial for biogeochemical processes. In contrast, members of *Phycisphaera* may represent specialized heterotrophic microbiota adapted to specific marine habitats, potentially limited to environments like AS (Sriswasdi et al. 2017).

The metabolic interactions of *Roseibium* and *Phycisphaera* with marine red algae were investigated through the genomic analyses of a *Roseibium* isolate (RMAR6-6), a *Phycisphaera* MAG (MAG 12) and a marine red alga (*P. purpureum* CCMP1328). Moreover, the metabolic interactions of *Roseibium* with marine red algae were tested by the co-culture study of RMAR6-6 and *P. purpureum* CCMP1328. Phototrophic marine red algae are able to produce carbohydrates, proteins, lipids and various secondary metabolites (e.g., flavonoids) from CO_2_ using light energy in the ocean and provide them to algae-associated heterotrophic bacteria (Cirri and Pohnert 2019; Seymour et al. 2017). Also, the transcriptomic analysis showed that genes involved in glycoside hydrolysis, oxidative stress and xenobiotic detoxification in RMAR6-6 were upregulated in the co-culture with *P*. *purpureum* CCMP1328 (Fig. 7C).

Nitrogen source serves as a growth-limiting factor for marine algae in the ocean. Moreover, nitrogen fixation by bacteria or cyanobacteria stands as a crucial source of fixed nitrogen for the growth of marine algae (Christie-Oleza et al. 2017; Zehr and Capone 2020; Zehr et al. 2016). The genomic analysis showed that, unlike *P. purpureum* CCMP1328, *Roseibium* RMAR6-6 has the ability to fix nitrogen and thus may provide nitrogen sources for the growth of marine red algae, which was confirmed by the co-culture experiments of *Roseibium* RMAR6-6 and *P. purpureum* CCMP1328 (Fig. 6). In addition, *P. purpureum* CCMP1328 has the ability to synthesize several B vitamins (B1, B2, B5–B7, and B9) but not vitamin B12, which is an essential cofactor for the growth of marine algae without cobalamin-producing ability in the ocean (Cooper et al. 2019; Croft et al. 2005; Rambo et al. 2020). *Roseibium* RMAR6-6 has the ability to synthesize cobalamin as well as other B vitamins. Moreover, the gene (*cobP*) involved in cobalamin synthesis was upregulated in the co-culture with *P. purpureum* CCMP1328 (Fig. 7C), suggesting that *Roseibium* is a likely source of bioavailable cobalamin for marine red algae. In the co-culture of *Roseibium* RMAR6-6 and *P. purpureum* CCMP1328, genes involved in the synthesis of thiamine (*thiE*), folate (*folC*), and pantothenic acid (*panC*) in RMAR6-6 were downregulated in the co-culture, whereas those in *P. purpureum* CCMP1328 were upregulate. This suggests that marine red algae may provide thiamine, folate, and pantothenic acid to algae-associated heterotrophic bacteria in the co-culture, although heterotrophic bacteria have the ability to produce them.

Microbes associated with marine algae are more susceptible to oxidative stress due to exposure to light than microbes in dark conditions. Genes involved in oxidative stress responses in RMAR6-6 were highly upregulated in the co-culture with *P. purpureum* CCMP1328 (Fig. 7C). DMSP, the most abundant organosulfur molecule in the ocean, that can act as an osmolyte, cryoprotectant, antioxidant, and sulfur and energy source in various marine organisms is purported to be produced mostly by marine algae (Strom et al. 2003; Sunda et al. 2002; Thume et al. 2018). However, recently it has been reported that notable amounts of DMSP are also produced by marine bacteria (Curson et al. 2017, 2018). RMAR6-6 has the ability to produce DMSP, which may act as an antioxidant (Fig. 4). Genes associated with DMSP synthesis were upregulated in the co-culture with RMAR6-6 (Fig. 7C), suggesting that DMSP production by *Roseibium* may protect *Roseibium* cells from oxidative stress caused by light exposure. In addition, genes involved in oxidative stress responses in *P. purpureum* CCMP1328 were also highly upregulated in the co-culture with RMAR6-6 (Fig. 7D), which suggests that *Roseibium* may exert a role in protecting marine red algae from oxidative and salt stresses by inducing genes involved in the stress responses of marine red algae. Also, previous studies reported that *Roseibium* representatives could alleviate various stresses, including oxidative and thermal stress, in corals (Camp et al. 2020; Dungan et al. 2021).

**Fig. 8:**
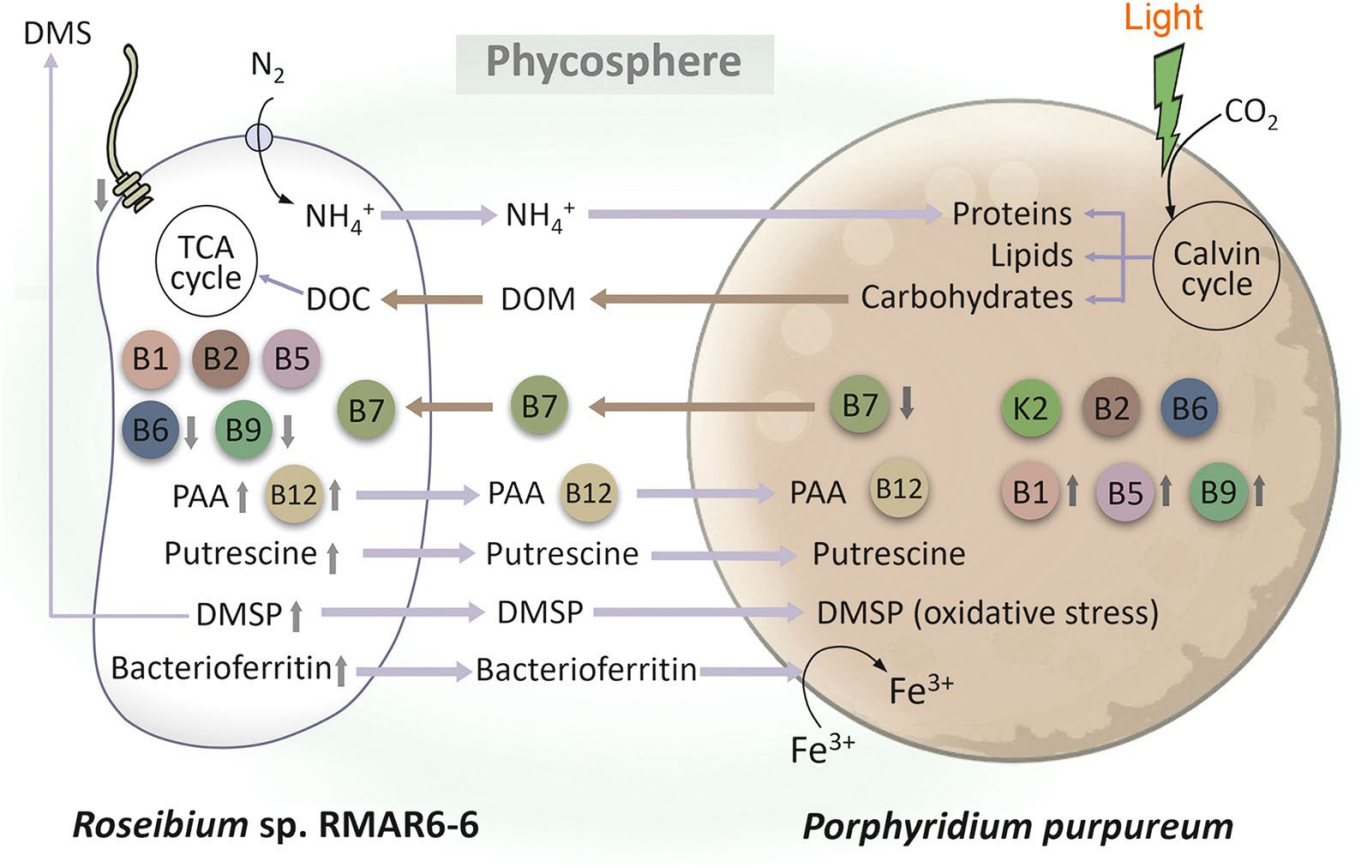
Potential metabolic relationships between a marine red alga, *Porphyridium purpureum* CCMP1328, and potential alga-associated bacterium, *Roseibium* RMAR6-6, predicted by genomic and transcriptomic analyses. Arrows beside metabolic mediators indicate their up- or downregulation in the co-culture of *P. purpureum* and *Roseibium* RMAR6-6 relative to monocultures. DMSP, dimethylsulfoniopropionate; DMS, dimethylsulfide; PAA, phenylacetate; DOC, dissolved organic carbon

Iron, an essential element involved in photosynthesis and respiration (Shaked and Lis 2012), is a limiting factor for the growth of marine algae because of its low concentration and poor solubility in seawater (Tortell et al. 1999). To facilitate iron acquisition in the ocean, many marine bacteria synthesize siderophores and Fe^3+^-siderophore transport systems (Kramer et al. 2020; Vraspir and Butler 2009). Moreover, because siderophores are synthesized by only a few eukaryotic phytoplankton, siderophore synthesis by bacteria may help marine algae acquire iron more efficiently (Rambo et al. 2020). RMAR6-6 has the capability to produce bacterioferritin, enhancing iron availability, which could be a vital metabolic interaction for promoting the growth of marine red algae in the ocean. Additionally, polyamines, including putrescine, are known to positively impact photosynthesis, cell proliferation and growth while providing protection against abiotic stresses in marine algae (Lin and Lin 2018; Xu et al. 2021). RMAR6-6, with its ability to produce putrescine, may contribute to the growth enhancement or stress alleviation in marine red algae. RMAR6-6 demonstrated the ability to produce PAA and 2-hydroxy-PAA (Fig. 5B), which are recognized as phytohormones known for promoting development, enhancing growth and improving stress tolerance in marine algae (Böttger et al. 2012). Overall, *Roseibium* RMAR6-6, which is capable of producing bacterioferritin, putrescine and PAA, has the potential to improve marine algae growth (Fries 1977; Kramer et al. 2020). Moreover, the upregulation of genes encoding PAA and bacterioferritin in co-culture with *P. purpureum* CCMP1328 (Fig. 7C) suggests that *Roseibium* may play a role in promoting the growth of marine algae by producing PAA and siderophores. Figure 8 illustrates the potential symbiotic relationships between *Roseibium*, a marine algae-associated bacterium, and *P. purpureum*, a marine red alga.

Previous metagenomic analyses indicated that *Phycisphaera* representatives associated with marine phototrophs may contribute to the growth or survival of marine phototrophs by removing reactive oxygen species, providing some vitamins and producing antibiotics (Rambo et al. 2020; Zheng et al. 2020). Our bioinformatic analysis of MAG 12 showed also that *Phycisphaera* members associated with marine red algae could have the ability to produce several B vitamins, some polyamines and bacitracin (an antibiotic) (Fig. 3B). However, it is not known whether these metabolic characteristics of *Phycisphaera* contribute to the growth or survival of marine red algae because these organisms also have the ability to synthesize B vitamins. Also, our analysis showed that *Phycisphaera* (MAG 12) may have limited metabolic versatility and biosynthetic capability compared to *Roseibium* RMAR6-6 (Fig. 3A, B), suggesting that *Phycisphaera* members may be highly dependent on marine phototrophs, which could be the major reason for the failure to isolate *Phycisphaera* representatives. However, bacitracin produced by *Phycisphaera* may play an important role in modulating the microbiome of marine phototrophs, possibly contributing to shaping healthy symbiotic populations in marine red algae (Ismail et al. 2016).

While *Roseibium* RMAR6-6 plays a crucial role as a symbiotic microbiota influencing the growth and survival of marine red algae, it is essential to recognize that it may represent just one aspect of the intricate network of bacteria involved in metabolic interactions with marine red algae. The marine algal environment likely hosts a multitude of symbiotic bacteria. RMAR6-6 possesses the ability to synthesize important growth factors such as vitamins and PAA, thereby enhancing the growth of marine algae. Notably, marine algae reciprocate by providing essential nutrients and carbon sources vital for the growth of RMAR6-6, sourced from either photosynthesis or cellular materials. Consequently, the growth and survival of RMAR6-6 may be inherently more contingent on the growth of marine red algae, emphasizing the potential dependence of *Roseibium* on marine algae rather than the reverse. In the co-culture with *P. purpureum* CCMP1328, numerous genes in RMAR6-6 could exhibit differential expression, while only a limited subset of genes in *P. purpureum* CCMP1328 may undergo differential expression in the presence of RMAR6-6 (Fig. 7).

In this study, we conducted microbial community analyses using nine marine red algal cultures cultivated in the laboratory. While laboratory propagation and culture were necessary to obtain sufficient algal cells for microbial community and metagenomic analyses, the inherent biases introduced through this laboratory propagation may not accurately reflect the true microbial communities of marine red algae in their natural oceanic environment. Therefore, it is necessary to study the metabolic relationships between the microbiome and marine algae more comprehensively to understand microbe-marine algae interactions in the ocean.

## Conclusion

Symbiotic relationships between marine algae and heterotrophic bacteria are important to affect the growth and survival of each other, but algae-associated bacteria and their metabolic interactions with marine algae have not been clearly elucidated. In this study, we analyzed the microbiota of nine marine red algal cultures, and identified *Roseibium* and *Phycisphaera* members as potential microbiota that may symbiotically interact with marine algae. The genomic analysis and co-culture and transcriptomic studies suggest that *Roseibium* RMAR6-6 may contribute to the growth or survival of marine algae through metabolic interactions. Our findings in this study will help to widen and deepen the understanding of the metabolic relationships between marine algae and symbiotic heterotrophic bacteria in the ocean.

### Supplementary Information

Below is the link to the electronic supplementary material.

Supplementary file1 (DOCX 1100 KB)

## Data Availability

The sequencing data of bacterial 16S rRNA-based metagenome, shotgun metagenome, and transcriptomes derived in this study have been deposited in the NCBI Sequence Read Archive under accession numbers SRS9357726, SRS9355529, SRS9461353, SRS9355858, and SRS9357153 (listed in BioProject, accession number: PRJNA742149). The whole genome sequences of strain RMAR6-6 and MAG 12 have been deposited in GenBank under the accession numbers CP019630-2 and CP098402, respectively.
