# Supplementary material for: Metabolic relationships between marine red algae and algae-associated bacteria
Source: Mar Life Sci Technol. 2024 May 8;6(2):298–314. doi: 10.1007/s42995-024-00227-z (PMC11136935; doi:10.1007/s42995-024-00227-z)
Supplement: Supplementary file 1 — Supplementary file1 (DOCX 1100 KB) [file 42995_2024_227_MOESM1_ESM.docx]

**Supplementary materials**

**Fig. S1** Maximum likelihood tree based on the ribulose-1,5-bisphosphate carboxylase gene (*rbcL*) sequences showing the phylogenetic positions of marine red algal cultures (highlighted in bold) used in this study. The *rbcL* sequences of the family *Cyanidiophyceae* were used as the outgroup and the tree was constructed and visualized using IQ-TREE software (Nguyen et al., 2015). Bootstrap values for 1,000 replicate analyses (>70%) are shown at branching points. Bar: 0.05 changes per nucleotide.


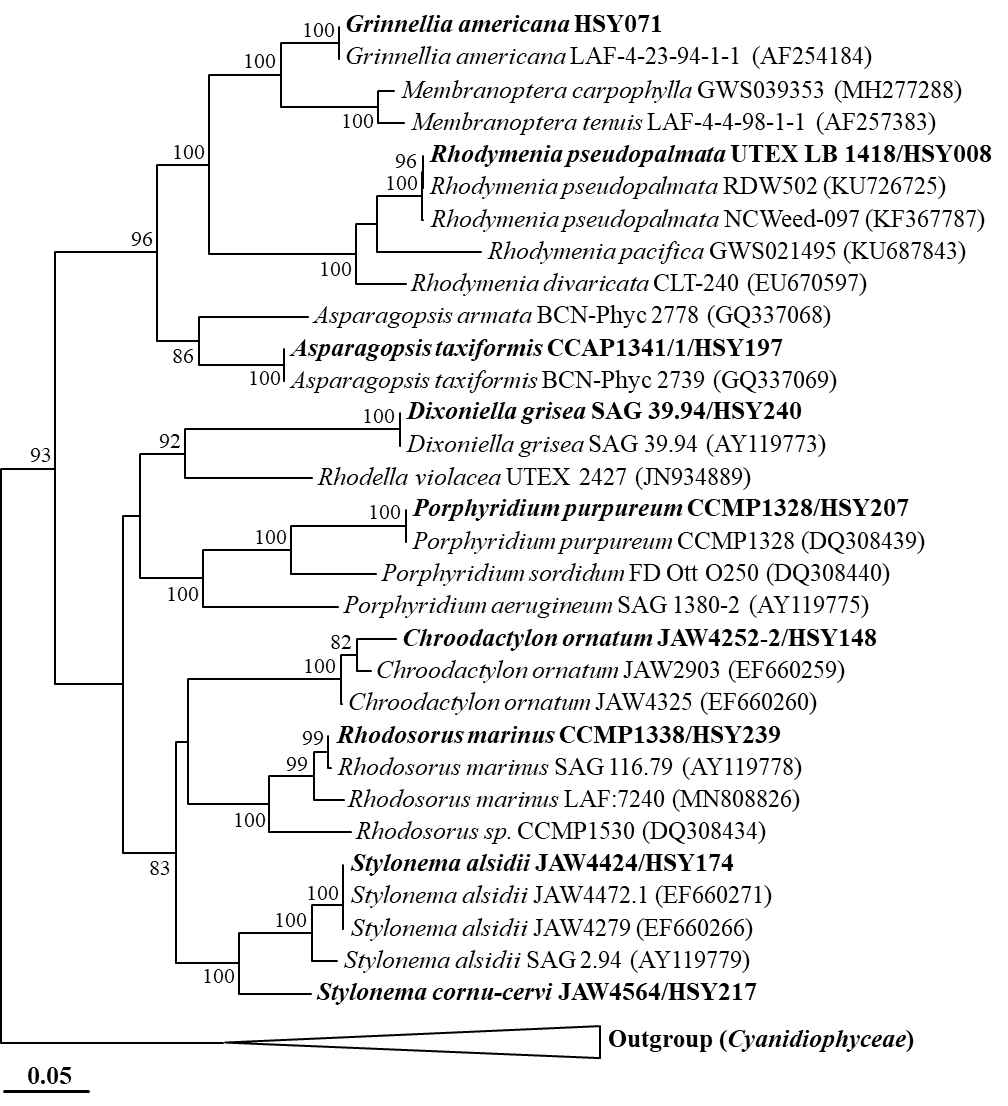


**Fig. S2** Bacterial taxonomic compositions in the bulk solution (**A**) and algal sphere regions (**B**) of marine red algae at the phylum level. At, *Asparagopsis taxiformis*; Ga, *Grinnellia americana*; Rp, *Rhodymenia pseudopalmata*; Dg, *Dixoniella grisea*; Pp, *Porphyridium purpureum*; Co, *Chroodactylon ornatum*; Rm, *Rhodosorus marinus*; Sa, *Stylonema alsidii*; Sc, *Stylonema cornu-cervi*. Bacterial community of seawater sampled from the East Sea of South Korea was analyzed as a reference.


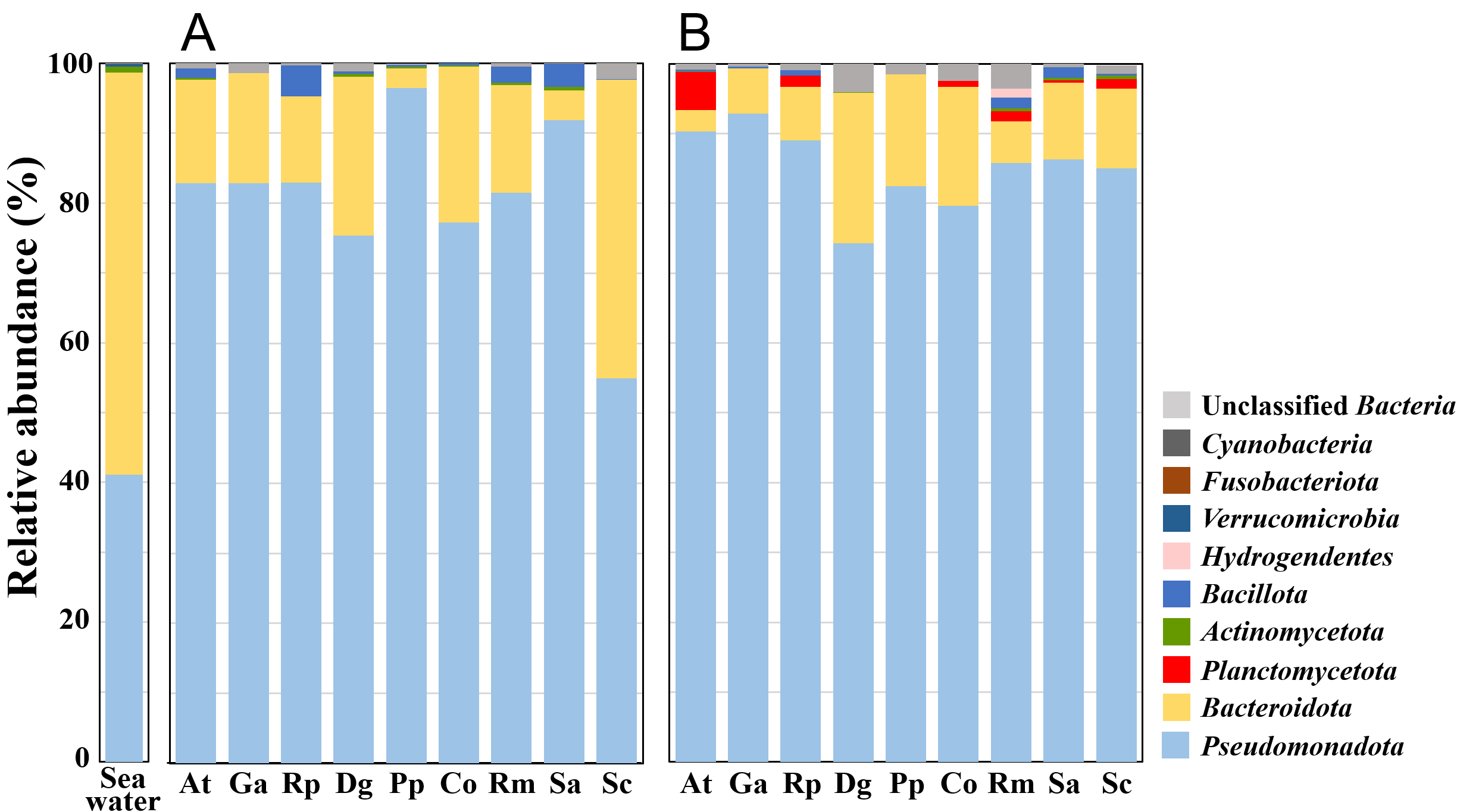


**Fig. S3** **A** Phylogenetic dendrogram of marine red algae based on *rbcL* sequences. Clustering dendrograms of bacterial communities in the bulk solution (**B**) and algal sphere (**C**) of marine red algae at the genus level.

**
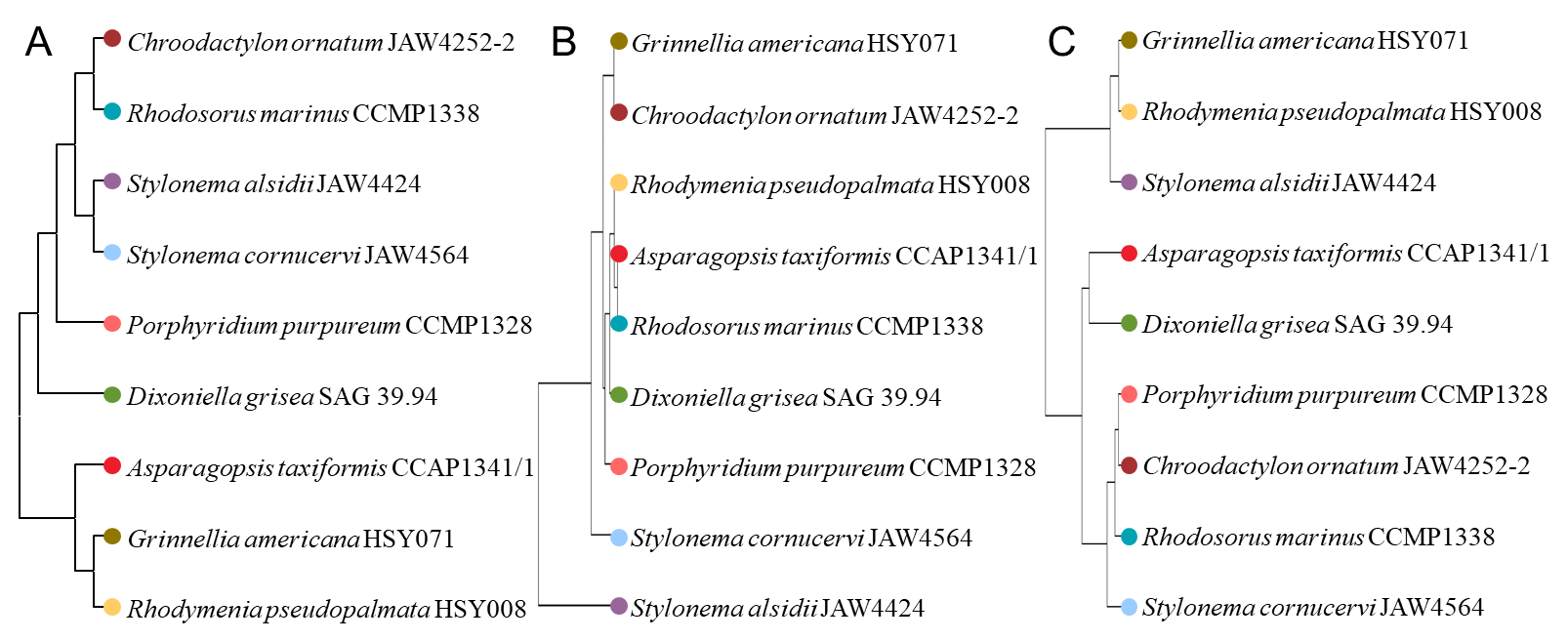
**

**Fig. S4** Graphical maps of the chromosome and two plasmids of *Roseibium* sp. RMAR6-6 generated by the CGView tool. Forward- and reverse-strand protein-coding sequences are indicated on the outermost two circles (differently colored by COG categories), respectively. G+C contents (black) and GC skews (+, green; –, magenta) are displayed on the third and fourth outermost circles, respectively.


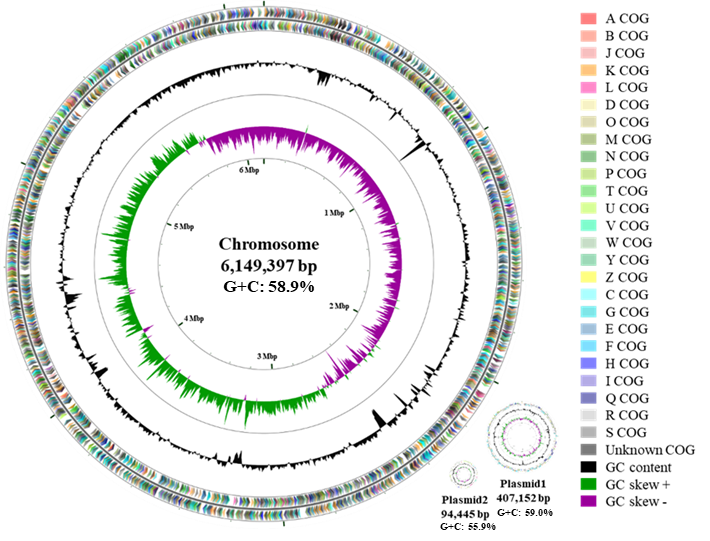


**Fig. S5.** LC-Q-TOF-MS ion chromatograms showing the production of riboflavin (**A**) and pyridoxine (**B**) by *Roseibium* sp. RMAR6-6.

**
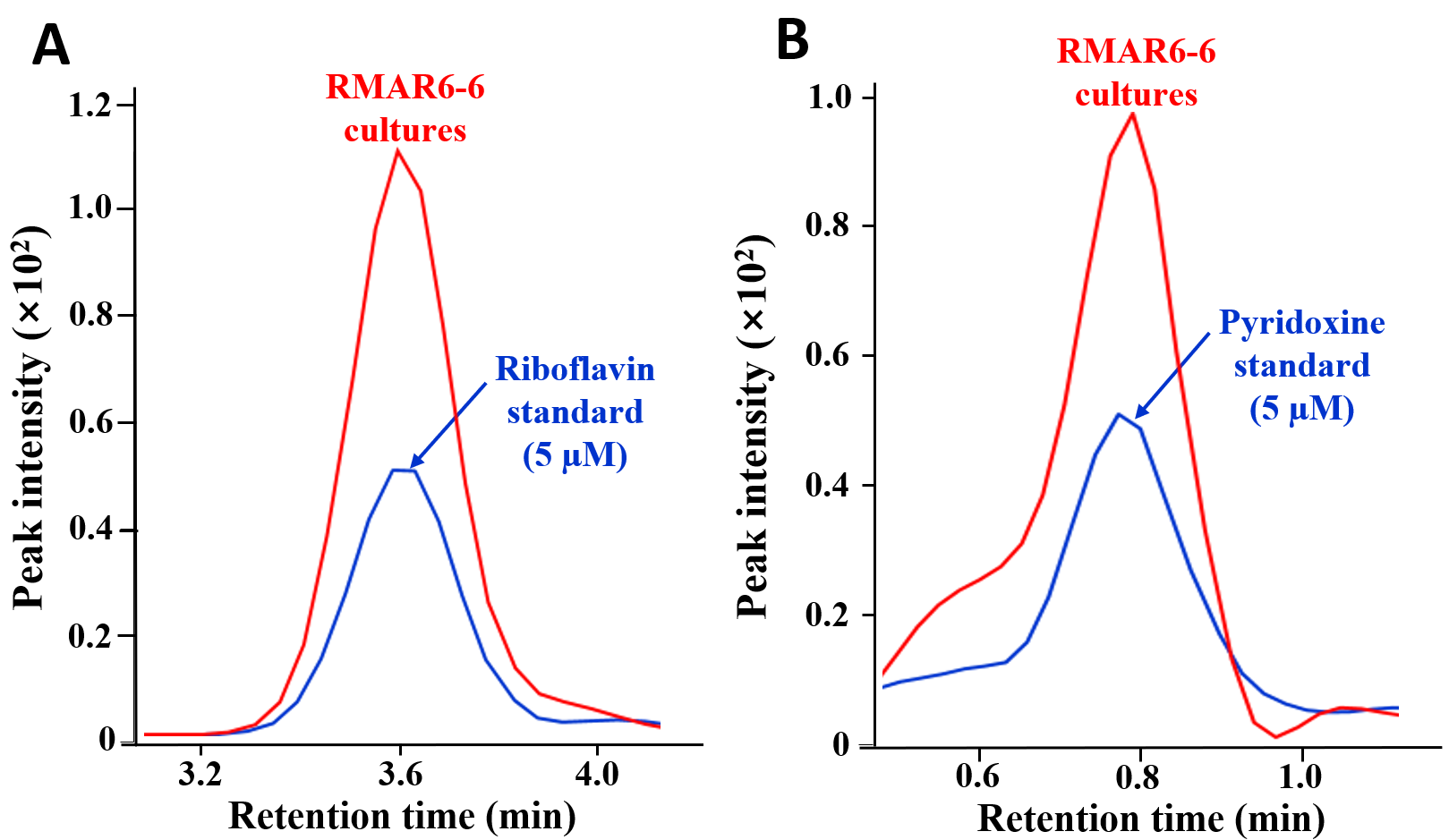
**

**Fig. S6** Scatter plots showing the relative transcriptional profiles of (**A**) *Roseibium* sp. RMAR6-6 and (**B**) *P. purpureium* CCMP1328 in mono- and co-culture. Dots indicate coding sequences (CDS) of strains RMAR6-6 and CCMP1328. Green lines indicate regression lines between the two cultures, and *R* values represent their Pearson correlation coefficients. Dotted lines represent cutoffs for two-fold differential expressions of CDS between the two cultures.

**
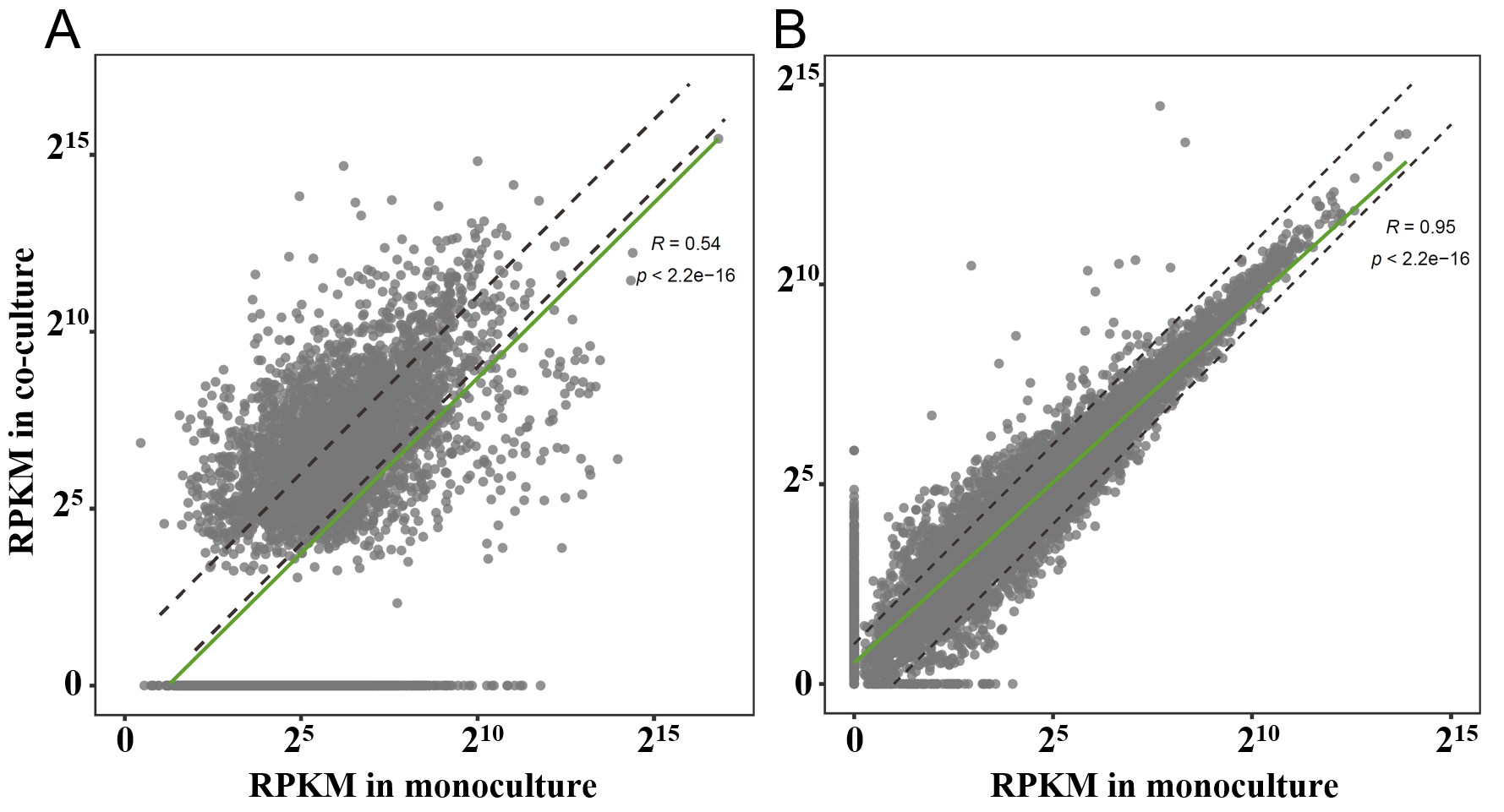
**

| Culture status | Taxon | Strain name | Algal type | Isolation place and culture collection center or collector |
| --- | --- | --- | --- | --- |
| Xenic | *Asparagopsis taxiformis* | CCAP1341/1 | Multicellular | Capo Posillipo, Naples, Italy; Culture Collection of Algae and Protozoa |
|  | *Grinnellia americana* | HSY071 | Multicellular | West Boothbay Harbor, ME, USA; Dr. Hwan Su Yoon |
|  | *Rhodymenia pseudopalmata* | UTEX LB 1418 | Multicellular | Unknown; UTEX Culture Collection of Algae at the University of Texas at Austin |
|  | *Dixoniella grisea* | SAG 39.94 | Unicellular | Yorktown, Virginia, USA; The Culture Collection of Algae at Göttingen University (SAG) |
|  | *Porphyridium purpureum* | CCMP1328 | Unicellular | Woods Hole, MA, USA; National Center for Marine Algae and Microbiota at Bigelow Laboratory |
|  | *Chroodactylon ornatum* | JAW4252-2 | Unicellular | Mallacoota Caravan Park, Victoria, Australia; Dr. John A. West |
|  | *Rhodosorus marinus* | CCMP1338 | Unicellular | Gif sur Yvette, France; National Center for Marine Algae and Microbiota at Bigelow Laboratory |
|  | *Stylonema alsidii* | JAW4424 | Multicellular | Point Lonsdale, Victoria, Australia; Dr. John A. West |
|  | *Stylonema cornu-cervi* | JAW4564 | Multicellular | Ubara, Katsuura, Chiba, Japan; Dr. Norio Kikuchi |
| Axenic | *Porphyridium purpureum* | CCMP1328 | Unicellular | Woods Hole, MA, USA; National Center for Marine Algae and Microbiota at Bigelow Laboratory |

**Table S1** Information on marine red algal cultures used in this study.

**Table S2** Bacterial strain, plasmid, and PCR primers used for the construction of a *fixH* gene knockout mutant RMAR6-6 *∆fixH* from *Roseibium* sp. RMAR6-6.

| Strain, plasmid, or primer | Description or sequence (5' → 3') | Source or reference(s) |
| --- | --- | --- |
| Bacterial strain |  |  |
| *Escherichia* *coli* BL21 (DE3) | F^–^ *dcm ompT* *hsd*S(r_B_^–^ m_B_^–^) *gal* λ (DE3) | Novagen |
| Plasmid or vector |  |  |
| pK18*mobsacB* | Integration vector; Km^r^ Neo^r^ *oriV_Ec_ oriT sacB* | Schäfer *et al*., 1994 |
| pK18*mobsacBΔfixH* | Carries a 2 bp deletion of *fixH* into pK18*mobsacB* | This study |
| PCR primers^†^ |  |  |
| fixH1F | CCCAAGCTTATGACGATGGTTCAA; *Hind*III | Knockout mutation of *fix* gene (FixH family protein) |
| fixH1R | GGCCGCGATTTCCTGGTTAGGCCTGT |  |
| fixH2F | ATGAAGCCGGACAGGCCTAACCAGGA |  |
| fixH2R | GCTCTAGATCAGTTCTTGACGAA; *Xba*I |  |
| fixGF | CCAAAGACACGTTCAAGGC | Confirmation of single crossover recombination |
| lacZR | ATCGCCATTCGCCATTCAGG |  |

^†^Restriction enzyme sites are underlined. F, forward; R, reverse.

**Table S3** Summary of taxonomic and genomic features of metagenomic assembled genomes (MAGs) obtained from the xenic culture of a marine red alga *Rhodosorus marinus.*

| MAG no. | Taxonomic hierarchy^†^ | Size (Mb) | No. of contigs | No. of genes | Complete-ness (%)^*^ | Contaminat-ion rate (%)^*^ |
| --- | --- | --- | --- | --- | --- | --- |
| 1 | *Bacteria*; *Pseudomonadota*; *Alphaproteobacteria*; *Rhodobacterales*; *Rhodobacteraceae*; *Ahrensia* | 3.57 | 84 | 3513 | 90.4 | 10.8 |
| 2 | *Bacteria*; *Pseudomonadota*; *Alphaproteobacteria*; *Rhodobacterales*; *Hyphomonadaceae*; *Oceanicaulis* | 3.19 | 56 | 3041 | 86.8 | 13.3 |
| 3 | *Bacteria*; *Pseudomonadota*; *Alphaproteobacteria*; *Rhodobacterales; Rhodobacteraceae* | 3.84 | 27 | 3816 | 99.6 | 0.8 |
| 4 | *Bacteria*; *Pseudomonadota*; *Alphaproteobacteria*; *Rhodobacterales*; *Rhodobacteraceae*; *Roseobacter* | 4.19 | 9 | 4179 | 98.9 | 1.5 |
| 5 | *Bacteria*; *Pseudomonadota*; *Alphaproteobacteria*; *Rhodobacterales*; *Rhodobacteraceae*; *Roseovarius*; *Roseovarius confluentis* | 4.58 | 442 | 4587 | 85.5 | 10.8 |
| 6 | *Bacteria*; *Pseudomonadota*; *Alphaproteobacteria; Sphingomonadales*; *Sphingomonadaceae* | 3.39 | 11 | 3284 | 98.8 | 0.6 |
| 7 | *Bacteria*; *Pseudomonadota*; *Gammaproteobacteria* | 5.11 | 27 | 4498 | 100.0 | 1.7 |
| 8 | *Bacteria*; *Pseudomonadota*; *Gammaproteobacteria*; *Nevskiales*; *Algiphilaceae*; *Algiphilus* | 3.08 | 337 | 2973 | 92.0 | 1.7 |
| 9 | *Bacteria*; *Pseudomonadota*; *Gammaproteobacteria* | 3.93 | 1,000 | 3834 | 76.8 | 2.2 |
| 10 | *Bacteria*; *Pseudomonadota*; *Gammaproteobacteria*; *Alteromonadales*; *Alteromonadaceae; Marinobacter*; *Marinobacter shengliensis* | 4.14 | 9 | 3782 | 98.8 | 0.7 |
| 11 | *Bacteria*; *Pseudomonadota*; *Gammaproteobacteria*; *Alteromonadales*; *Alteromonadaceae*; *Marinobacter*; *Marinobacter salaries* | 5.13 | 397 | 4930 | 95.7 | 13.5 |
| 12 | *Bacteria*; *Planctomycetota*; *Phycisphaerales*; *Phycisphaerae* | 3.62 | 1 | 3120 | 84.2 | 1.4 |
| 13 | *Bacteria*; *Planctomycetota*; *Planctomycetia*; *Pirellulales*; *Pirellulaceae*; *Rhodopirellula* | 9.64 | 134 | 6732 | 90.1 | 12.6 |
| 14 | *Bacteria*; *Bacteroidetes*; *Flavobacteriia*; *Flavobacteriales*; *Flavobacteriaceae* | 5.18 | 288 | 4598 | 97.2 | 2.9 |
| 15 | *Bacteria*; *Bacteroidota*; *Flavobacteriia*; *Flavobacteriales*; *Flavobacteriaceae*; *Muricauda* | 3.26 | 4 | 3018 | 99.7 | 0.1 |
| 16 | *Bacteria*; *Cyanobacteria*; *Gloeoemargaritales*; *Gloeomargaritaceae* | 0.31 | 54 | 343 | 13.7 | 0.1 |
| 17 | *Archaea*; *Euryarchaeota*; *Thermoplasmata* | 1.78 | 5 | 1841 | 94.8 | 0.8 |

**^†^** Taxonomic hierarchy of the MAGs was inferred based on the GenBank taxonomic classification system in Genome Taxonomy Database.

**Table S4** Carbon utilization ability of *Roseibium* sp. RMAR6-6 tested using Biolog GN2 MicroPlate.

|  | Positive | Negative |
| --- | --- | --- |
| Carbon source | Glycogen, Tween 40, *N*-acetyl-d-glucosamine, adonitol, l-arabinose, d-arabitol, d-cellobiose, *i*-erythritol, d-fructose, l-fructose, d-galactose, gentiobiose, *α*-d-glucose, *m*-inositol, *α*-d-lactose, lactulose, maltose, d-mannitol, d-mannose, d-melibiose, *β*-methyl-d-glucoside, d-psicose, d-raffinose, l-rhamnose, d-sorbitol, sucrose, d-trehalose, turanose, xylitol, acetic acid, citric acid, d-galactonic acid lactone, d-galacturonic acid, d-gluconic acid, d-glucosaminic acid, d-glucuronic acid, *α*-hydroxybutyric acid, *β*-hydroxybutyric acid, *α*-keto butyric acid, *α*-keto glutaric acid, α-keto valeric acid, d,l-lactic acid, malonic acid, propionic acid, quinic acid, d-saccharic acid, succinic acid, succinamic acid, glucuronamide, l-alaninamide, d-alanine, l-alanine, l-asparagine, l-aspartic acid, l-glutamic acid, glycyl-l-aspartic acid, glycyl-l-glutamic acid, l-histidine, hydroxy-l-proline, l-ornithine, l-phenylalanine, l-proline, l-pyroglutamic acid, d-serine, l-serine, l-threonine, d,l-carnitine, γ-aminobutyric acid, uridine, thymidine, phenyethylamine, putrescine, 2,3-butanediol, d,l-*α*-glycerol phosphate, glucose-1-phosphate, glucose-6-phosphate | α-Cyclodextrin, dextrin, Tween 80, *N*-acetyl-d-galactosamine, pyruvic acid methyl ester, succinic acid mono-methyl ester, *cis*-aconitic acid, formic acid, *γ*- hydroxybutyric acid, *p*-hydroxy phenylacetic acid, itaconic acid, sebacic acid, bromo-succinic acid, l-alanylglycine, l-leucine, urocanic acid, inosine, 2-aminoethanol, glycerol |

**Table S5** Summary of Illumina sequencing reads of transcriptomes obtained from *Roseibium* strain RMAR6-6 and *Porphyridium purpureium* CCMP1238 in mono- and co-culture.

|  | Monoculture | |  | Co-culture | |
| --- | --- | --- | --- | --- | --- |
|  | Strain  RMAR6-6 | Strain  CCMP1238 |  | Strain RMAR6-6 | Strain  CCMP1238 |
| No. of total reads | 62236310 | 60000429 |  | 197793532 | |
| No. of high-quality sequencing reads mapped on the genome (%*) | 34600010 (55.59) | 35773814 (59.62) |  | 212248 (0.10) | 36541953 (18.47) |
| No. of sequencing reads mapped to coding sequences (%**) | 32302367  (93.36) | 1006657  (2.81) |  | 69091  (32.55) | 2743131  (7.50) |

***^,^ ****The numbers in parentheses indicate percentages of sequencing reads for total reads and high-quality sequencing reads, respectively.

**References**

Nguyen LT, Schmidt HA, von Haeseler A, Minh BQ (2015) IQ-TREE: a fast and effective stochastic algorithm for estimating maximum-likelihood phylogenies. Mol Biol Evol 32:268–274

Schäfer A, Tauch A, Jäger W, Kalinowski J, Thierbach G, Pȕhler A (1994) Small mobilizable multi-purpose cloning vectors derived from *Escherichia coli* plasmids pK18 and pK19: selection of defined deletions in the chromosome of *Corynebacterium glutamicum*. Gene 145:69–73
